# Ubiquitin‐specific protease 22 controls melanoma metastasis and vulnerability to ferroptosis through targeting SIRT1/PTEN/PI3K signaling

**DOI:** 10.1002/mco2.684

**Published:** 2024-08-12

**Authors:** Huiyan Sun, Yu Meng, Lei Yao, Songtao Du, Yayun Li, Qian Zhou, Yihuang Liu, Yating Dian, Yuming Sun, Xiaomin Wang, Xiao‐wei Liang, Guangtong Deng, Xiang Chen, Furong Zeng

**Affiliations:** ^1^ Department of Dermatology Xiangya Hospital Central South University Changsha China; ^2^ National Engineering Research Center of Personalized Diagnostic and Therapeutic Technology Changsha China; ^3^ Furong Laboratory Changsha China; ^4^ Hunan Key Laboratory of Skin Cancer and Psoriasis, Hunan Engineering Research Center of Skin Health and Disease, Xiangya Hospital Central South University Changsha China; ^5^ National Clinical Research Center for Geriatric Disorders (Xiangya Hospital) Changsha China; ^6^ Department of Breast Reconstruction Tianjin Medical University Cancer Institute and Hospital Tianjin China; ^7^ Department of Liver Surgery Xiangya Hospital Central South University Changsha China; ^8^ Department of Colorectal Surgical Oncology The Tumor Hospital of Harbin Medical University Harbin China; ^9^ Department of Dermatology The Third Xiangya Hospital Central South University Changsha China; ^10^ Department of Plastic and Cosmetic Surgery Xiangya Hospital Central South University Changsha China; ^11^ Department of Breast Surgery Xiangya Hospital Central South University Changsha China; ^12^ Department of Oncology Xiangya Hospital Central South University Changsha China

**Keywords:** ferroptosis, melanoma, metastasis, topotecan, USP22

## Abstract

Metastasis is a major contributing factor that affects the prognosis of melanoma patients. Nevertheless, the underlying molecular mechanisms involved in melanoma metastasis are not yet entirely understood. Here, we identified ubiquitin‐specific protease 22 (USP22) as a pro‐oncogenic protein in melanoma through screening the survival profiles of 52 ubiquitin‐specific proteases (USPs). USP22 demonstrates a strong association with poor clinical outcomes and is significantly overexpressed in melanoma. Ablation of USP22 expression remarkably attenuates melanoma migration, invasion, and epithelial–mesenchymal transition in vitro and suppresses melanoma metastasis in vivo. Mechanistically, USP22 controls melanoma metastasis through the SIRT1/PTEN/PI3K pathway. In addition, we conducted an United States Food and Drug Administration‐approved drug library screening and identified topotecan as a clinically applicable USP22‐targeting molecule by promoting proteasomal degradation of USP22. Finally, we found that both pharmacological and genetic silence of USP22 sensitize RSL3‐induced ferroptosis through suppressing the PI3K/Akt/mTOR pathway and its downstream SCD, and ferroptosis inhibitor could partly rescued the decreased lung metastasis by topotecan in vivo. Overall, our findings reveal a prometastatic role of USP22 and identify topotecan as a potent USP22‐targeting drug to limit melanoma metastasis.

## Graphical abstract

Ubiquitin‐specific protease 22 (USP22) stabilizes SIRT1 and activates the PI3K/Akt/mTOR pathway through enhancing SIRT1‐mediated PTEN deacetylation, thereby promoting melanoma metastasis both in vitro and in vivo. Pharmacological or genetic inhibition of USP22 sensitizes ferroptosis by suppressing the PI3K/Akt/mTOR pathway and its downstream target SCD. Furthermore, our study identified topotecan as a clinical USP22‐targeting drug capable of effectively suppressing melanoma metastasis in vivo.

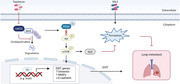

## 1 INTRODUCTION

Melanoma is one of the deadliest skin cancers, with an estimated 57,000 new deaths occurring worldwide each year.^1^ Metastatic dissemination is a pivotal determinant of poor prognosis, and the 5‐year survival rate of metastatic melanoma patients is merely 32%.^2^, ^3^, ^4^ Although immunotherapy (anti‐PD‐1 or anti‐CTLA‐4 antibodies) and targeted therapy (BRAF/MEK inhibitors) have been approved for the treatment of advanced melanoma patients, only a minority of patients derive benefit.^5^, ^6^ Targeted therapy often links to inevitable drug resistance, while immunotherapy encounters low efficiency due to heterogenous response.^7^ Consequently, it remains imperative to identify robust biomarkers and investigate novel approaches to improve outcomes for patients with advanced melanoma.

Ubiquitin‐specific proteases (USPs), the largest subclass of deubiquitinases, hold significant implications in modulating cancer biology.^8^ USPs have been involved in a variety of metastasis‐related biological processes, such as the activation of epithelial–mesenchymal transition (EMT) and resistance to cell death.^9^, ^10^ For example, USP4 enhances the invasive and migratory capabilities of melanoma cells by facilitating EMT.^11^ Moreover, depletion or pharmacological inhibition of USP7 dampens cell migration and invasion and increases melanoma susceptibility to BRAF inhibitors.^8^, ^12^ Our group has also demonstrated that USP7 inhibition suppresses melanoma cell proliferation, migration, and invasion.^13^ However, the potential oncogenic role of other USPs in melanoma metastasis still need further investigations.

USP22, a member of the USP family, acts a pivotal role in the cancer‐related death signature and primarily regulates cancer progression through its deubiquitinase activity.^14^ As a key component of Spt–Ada–Gcn5 acetyltransferase complex (SAGA) complex, USP22 participates in the regulation of transcription factors during cancer progression by deubiquitinating histones (H2A and H2B).^15^ Furthermore, USP22 directly targets several oncogenes including BMI‐1^16^ and cyclin B1,^17^ independently of the SAGA complex. In melanoma, USP22 interacts with and deubiquitinates YAP, thereby favoring melanoma cell proliferation.^18^ Besides, USP22 in melanoma cells stabilizes STAT1 and promotes the interferon pathway, which improves the cytotoxic effects of CD8^+^ T cells.^19^ To date, these studies have focused on the impact of USP22 on melanoma growth and the interaction between melanoma cells and immune cells. However, a comprehensive understanding of the role and underlying mechanisms by which USP22 regulates melanoma metastasis is often overlooked and remains largely unknown.

Ferroptosis, an iron‐dependent form of regulated cell death, is closely related to oxidative stress.^20^, ^21^ Resistance to ferroptosis facilitates melanoma metastasis in lymph and blood, with reduced oxidative stress.^22^ Previously, we have summarized the regulatory role of USPs in ferroptosis, indicating that targeting ferroptosis by USPs presents a potential approach to suppress cancer progression.^21^ USP22 has been showed to protect pancreatic β cells and cardiomyocyte from ferroptosis.^23^, ^24^ However, the role of USP22 in ferroptosis susceptibility in melanoma remains elusive. In this study, we identified USP22 is overexpressed in melanoma and facilitates melanoma metastasis in vitro and in vivo. Mechanistically, USP22 controls melanoma metastasis by targeting SIRT1/PTEN/PI3K signaling axis. Through screening the library of United States Food and Drug Administration (US FDA)‐approved antitumor drugs, we further identified topotecan as a USP22‐targeting molecule capable of suppressing melanoma metastasis. Finally, we explored the relationship between USP22 and melanoma vulnerability to ferroptosis and found that USP22 inhibition sensitizes melanoma to RSL3‐induced ferroptosis through inhibiting PI3K/Akt/mTOR pathway‐mediated SCD expression. Altogether, our findings indicate that targeting USP22 represents a promising avenue to treat melanoma metastasis.

## 2 RESULTS

### 2.1 Upregulation of USP22 in melanoma and its correlation with poor clinical outcomes

**FIGURE 1:**
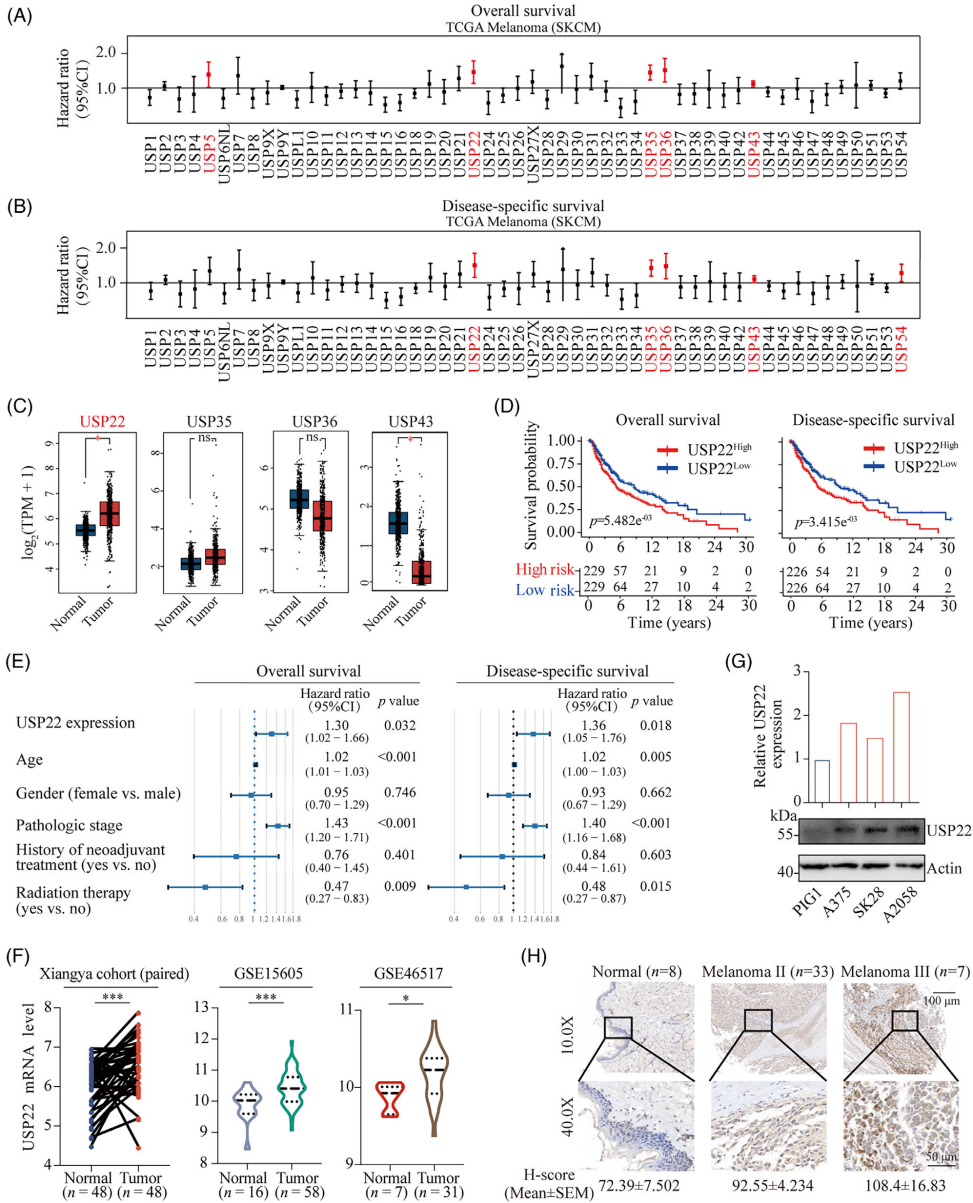
USP22 is overexpressed in melanoma and associated with a poor prognosis. (A and B) Univariate Cox regression analysis to reveal the association between the USP family and overall survival (A) or disease‐specific survival (B) of melanoma patients. (C) Bioinformatic analysis for the expression of USP22, USP35, USP36, USP43 expression in melanoma (*n* = 461) and normal skin tissues (*n* = 558). (D) Kaplan–Meier curves depicting overall survival or disease‐specific survival difference between USP22 high and USP22 low groups. (E) Forest plot showing the results of hazard ratio's (HR) from multivariate Cox regression analysis for the overall survival and disease‐specific survival of melanoma patients. (F) USP22 mRNA expression levels in human melanoma and normal skin data based on Xiangya cohort and two different GSE databases (GSE15605 and GSE46517). (G) USP22 protein levels in normal melanocytes (PIG1) and melanoma cell lines (A375, SK‐Mel‐28, and A2058). (H) Representative images of immunohistochemistry (IHC) staining of USP22 in tissue assay quantified with *H*‐score.

USPs, the main members of the deubiquitinase family, have been implicated as critical players in melanoma progression. To identify potential oncogenic candidates within the human USPs, we systematically analyzed the survival profiles of 52 USPs genes based on datasets from TCGA‐SKCM database. A univariate Cox regression analysis was performed for the primary screening of USPs. Using the condition of hazard ratio > 1, we demonstrated that USP22, USP35, USP36, and USP43 are significantly associated with poor overall survival and disease‐specific survival (Figure 1A,B). Notably, USP22 stands out as one of the most differentially expressed genes, with upregulated expression in melanoma compared with normal skin (Figures 1C and S1A). To further validate the association between USP22 expression and poor prognosis in melanoma, we performed Kaplan–Meier analysis and found that elevated USP22 expression is significantly linked with shortened overall survival and disease‐specific survival (Figure 1D). To define that USP22 is an independent risk factor associated with clinical outcomes, we conducted a multivariate Cox analysis based on available clinical information, including patient age, gender, pathological stage, and treatment, and substantiated USP22 expression as an independent prognostic factor (Figure 1E). Moreover, we found that USP22 expression is relatively elevated in melanoma when compared with normal skin or tumor‐adjacent normal tissues in Xiangya dataset and two different GSE datasets (GSE15605 and GSE46517) (Figures 1F and S1B). We also detected USP22 expression in melanoma cell lines and melanocyte (PIG1), with high expression in melanoma cells but relatively low expression in melanocytes (Figure 1G). To further verify USP22 protein levels in melanoma, we performed immunohistochemistry (IHC) staining and detected markedly higher intensities of USP22 immunostaining in melanoma compared with normal tissues (Figures 1H and S1C). Notably, USP22 protein levels were positively correlated with the pathological stage of melanoma (stage II, *H*‐score, 92.55 ± 4.234 vs. stage III, *H*‐score 108.4 ± 16.83) (Figures 1H and S1C). Together, our results demonstrated the upregulation of USP22 in melanoma and its correlation with poor clinical outcomes.

### 2.2 USP22 promotes melanoma metastasis both in vitro and in vivo

**FIGURE 2:**
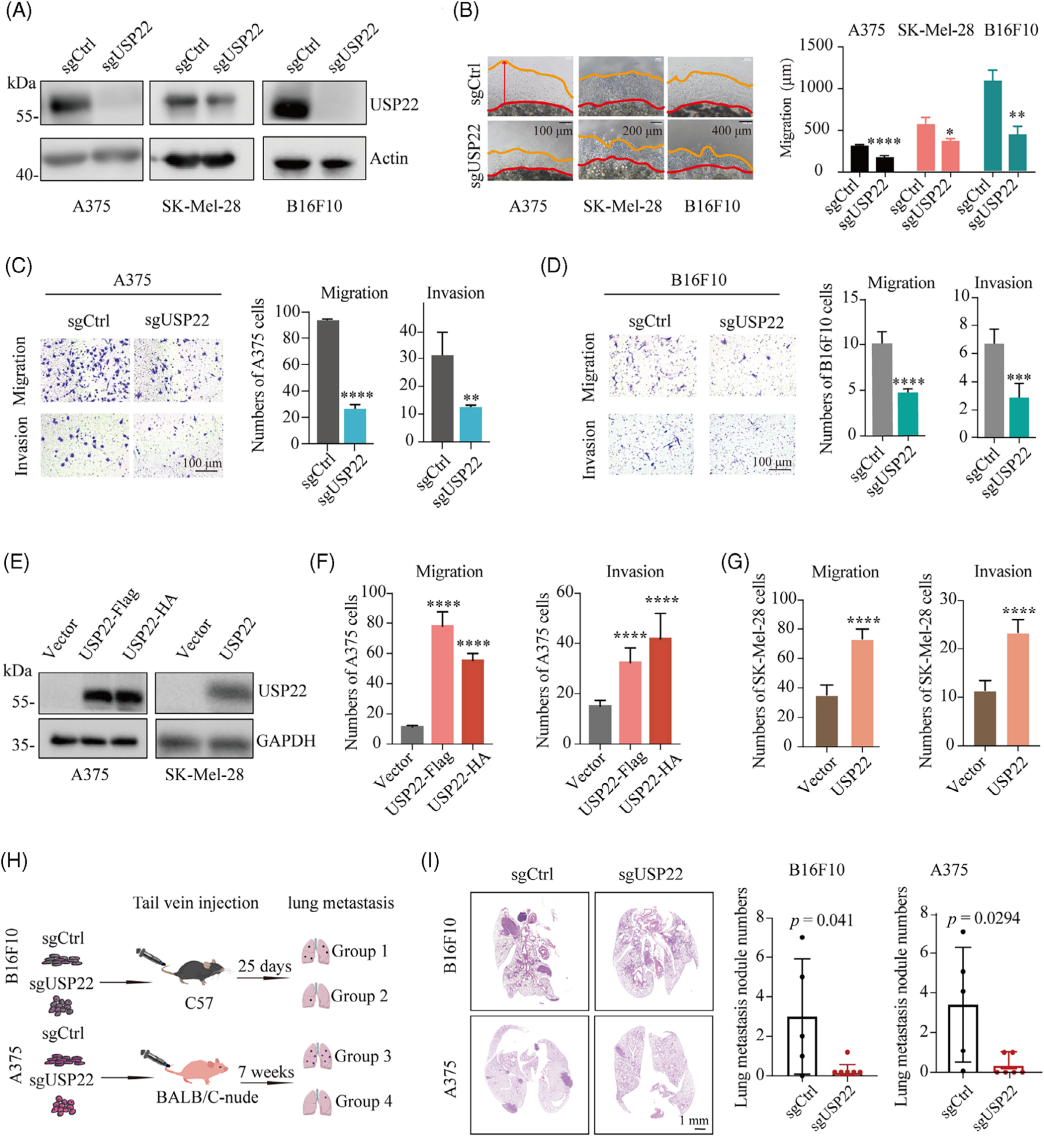
USP22 loss suppresses melanoma metastasis both in vitro and in vivo. (A) Western blot analysis of the indicated proteins in control (sgCtrl) and USP22‐knockout (sgUSP22) A375, SK‐Mel‐28, and B16F10 cells. (B) 3D Matrigel drop invasion assay for A375, SK‐Mel‐28, and B16F10 cells after USP22 knockout (sgUSP22). Indicated medium was exchanged every 3 days. The distances of migrated cells away from edge of the matrigel drop were measured as migration (µm) on Day 6. Experiments were triplicate repeated. Representative images and scare bars are shown. (C and D) Transwell assay quantifying the migration (without extracellular matrix) and invasive (with extracellular matrix) capacity for A375 and B16F10 cells after USP22 knockout (sgUSP22). Migrated and invaded cells were determined for 12−20 h. Five random areas were selected. (E) Western blot analysis of the indicated proteins in control (Vector) and USP22‐overexpressing A375 and SK‐Mel‐28 cells. (F and G) Transwell assay quantifying the migration and invasion for A375 (F) and SK‐Mel‐28 cells (G) after USP22 overexpression. (H and I) Schematic view and representative hematoxylin–eosin (H&E) images of lung metastasis of mice after tail vein injection of B16F10 or A375 cells with USP22‐knockout (sgUSP22) or control (sgCtrl) cells. Two‐tailed unpaired Student's *t*‐test was performed in (B–D, G, and I), and one‐way ANOVA was used in (F). *p < 0.05, **p < 0.01, ***p < 0.001, ****p < 0.0001.

To investigate the potential oncogenic mechanism of USP22, we initially generated USP22‐deficient melanoma cells via CRISPR–Cas9 technology (Figure 2A) and observed minimal suppression of cell proliferation, as detected by CCK‐8 assay (Figure S2A). We also constructed USP22‐overexpressing A375 and SK‐Mel‐28 melanoma cells and found that overexpression of USP22 exerts a negligible impact on melanoma cell proliferation (Figures 2E and S2B). To elucidate the role of USP22 in vivo, USP22‐deficient A375 cells were subcutaneously injected into the BALB/c nude mice (Figure S2C). We observed that USP22 knockout has a minimal effect on the growth of xenografted melanoma tumors (Figure S2C,D), and the bodyweight of mice is comparable between the USP22‐deficient and control groups (Figure S2E). These results suggested that USP22 facilitates melanoma progression via another mechanism.

Cell motility and migration play a crucial role in the progression of melanoma.^25^ To determine whether USP22 affects melanoma cell migration and invasion, we employed 3D Matrigel drop invasion,^26^ wound healing and transwell assays. The shorter migrated distance and delayed wound repair were detected in USP22‐deficient melanoma cells (Figures 2B and S3A,B). Similarly, the number of invasive melanoma cells was significantly reduced in both USP22‐deficient melanoma cells and USP22 knockdown cells with siRNA (Figures 2C,D and S3C). Conversely, USP22‐overexpressing melanoma cells exhibited an increased number of migrative and invasive melanoma cells compared with control cells (Figures 2E–G and S3D,E). Furthermore, we used a lung metastasis model in C57BL/6 mice to mimic the advanced stages of the metastatic process (Figure 2H). Mice that received injections of USP22‐deficient B16F10 cells exhibited a reduced metastatic burden, characterized by a lower number of lung micro‐metastases per lung section, as detected by hematoxylin–eosin (H&E) staining of tumor nodule specimens (Figure 2I). To eliminate the potential influence of the immune system on melanoma metastasis, we replicated the lung metastasis model in immunodeficient mice (Figure 2H). Consistently, H&E images showed that USP22 loss significantly impaired melanoma lung metastasis with less metastatic foci (Figure 2I). These findings suggested that USP22 promotes melanoma metastasis both in vitro and in vivo.

### 2.3 Elevated USP22 induces EMT activation in melanoma

**FIGURE 3:**
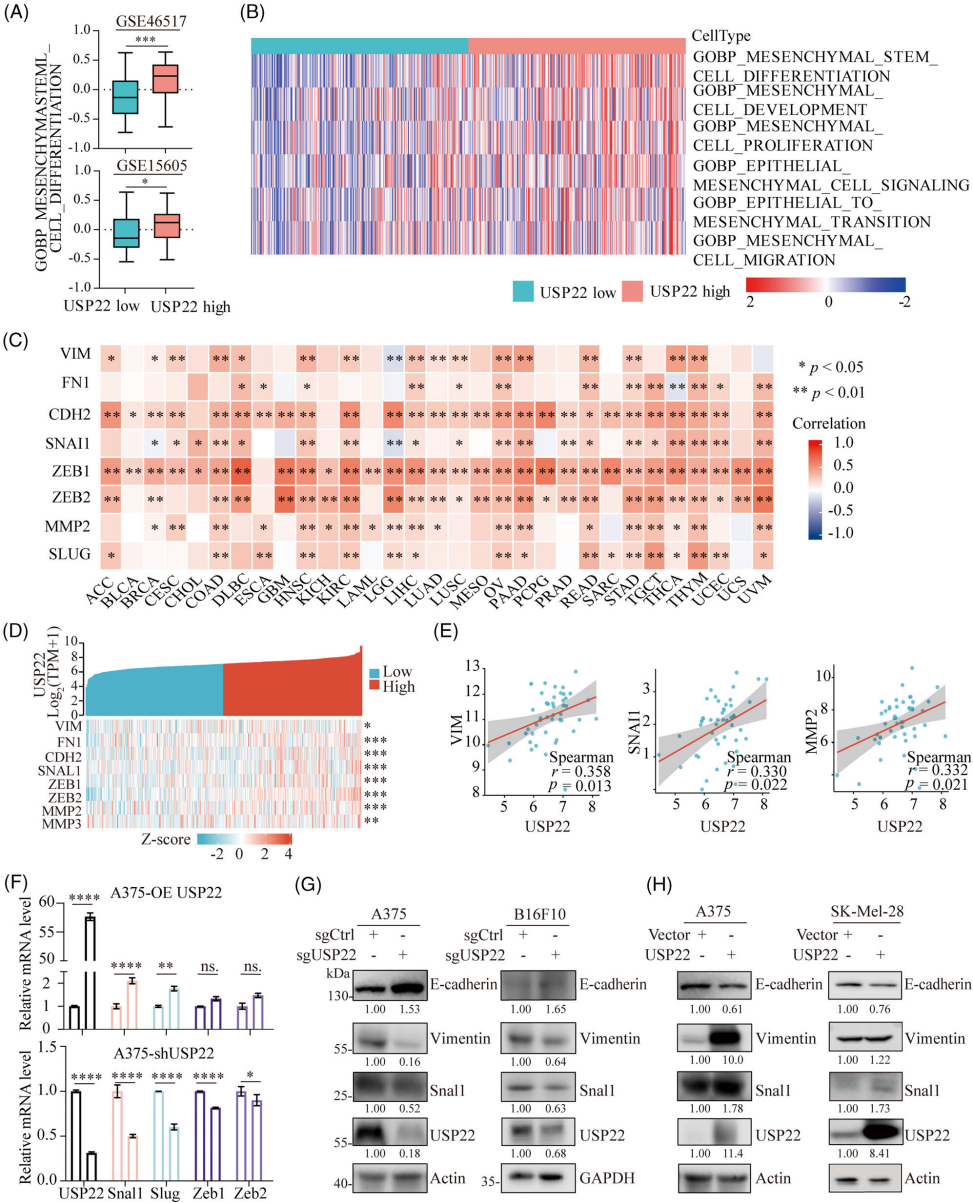
USP22 promotes EMT activation in melanoma. (A) Correlation between USP22 expression with mesenchymal stem cell differentiational pathway based on GSE46517 and GSE15605 databases. (B) Heatmap showing USP22 expression positively associated with epithelial–mesenchymal transition (EMT)‐related pathways in TCGA‐SKCM datasets. (C and D) Heatmap showing USP22 expression positively associated with EMT‐related genes in different cancers (C) including melanoma (D) in TCGA datasets. (E) Spearman's correlation between the expression of vimentin (VIM), SNAL1, MMP2, and USP22 based on data from Xiangya cohort. (F) The mRNA expression levels of EMT‐related transcription factors in USP22‐overexpressing and USP22‐knockdown (shUSP22) A375 cells. (G and H) The protein expression levels of EMT‐related markers including E‐cadherin, vimentin and snal1 in USP22‐knockout (sgUSP22) and USP22‐overexpressing (USP22) melanoma cells. *p < 0.05, **p < 0.01, ***p < 0.001.

Accumulating evidence indicates that melanoma cells enhance their metastatic capabilities by obtaining mesenchymal‐like properties.^27^, ^28^, ^29^ Hence, we hypothesized that USP22 might facilitate melanoma metastasis through promoting EMT. We firstly analyzed the pathway associated with mesenchymal stem cell differentiation based on GSE datasets (GSE15605 and GSE46517) and found that the mesenchymal stem cell differentiational pathway is significantly activated in melanoma cells with elevated USP22 expression (Figure 3A). Consistently, USP22 expression is positively associated with numerous EMT‐related pathways in TCGA‐SKCM datasets (Figure 3B). We further analyzed genes associated with EMT in TCGA datasets and found a positive correlation between USP22 and EMT‐related genes across various cancer types, including melanoma (Figure 3C,D). Vimentin, SNAL1, and MMP2 are essential genes for melanoma EMT. Therefore, we validated the association between USP22 and these three genes within the Xiangya melanoma datasets and found positive correlations between USP22 and EMT markers vimentin, SNAL1 and MMP2 (Figure 3E). To further unravel this aspect, we conducted RT‐PCR to evaluate the expression of EMT transcription factors (EMT‐TFs) in USP22 knockdown or overexpression A375 cells. Our results indicated that EMT‐TFs expressions are downregulated following USP22 knockdown and upregulated with USP22 overexpression (Figure 3F). Western blotting assay revealed elevated levels of the epithelial marker E‐cadherin protein and reduced levels of the mesenchymal markers vimentin and SNAL1 protein in USP22‐deficient melanoma cells (Figure 3G). Conversely, decreased E‐cadherin and increased vimentin and SNAL1 protein levels were detected in USP22‐overexpressing melanoma cells (Figure 3H). These results demonstrated that USP22 might promotes melanoma metastasis by inducing EMT activation.

### 2.4 USP22 potentiates melanoma metastasis and EMT through activating PI3K/Akt/mTOR pathway

**FIGURE 4:**
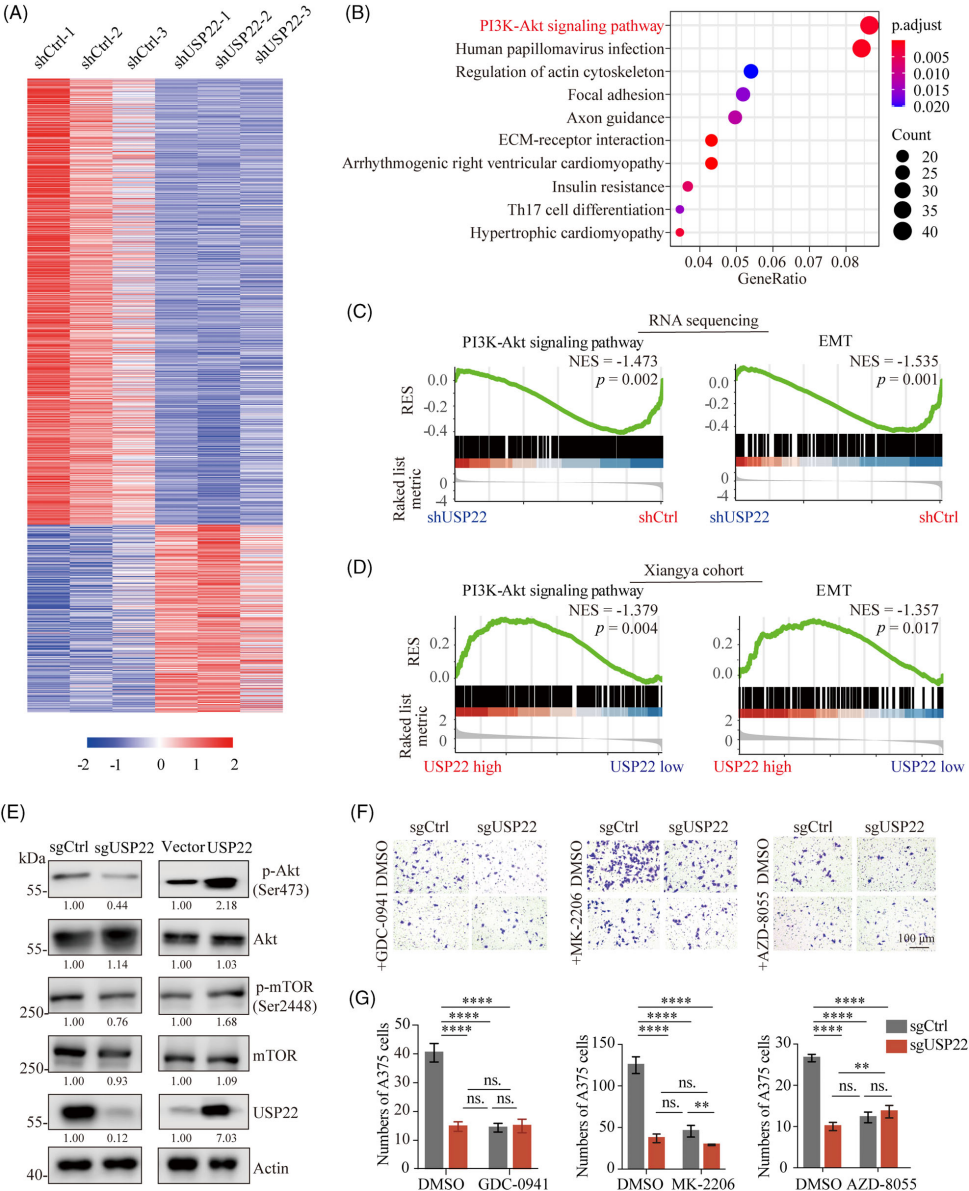
USP22 potentiates melanoma metastasis and EMT through activating PI3K/Akt/mTOR pathway. (A) Transcriptome sequencing was performed on USP22‐knockdown (shUSP22) and control (shCtrl) A375 cells and the results were visualized in a heatmap. (B) RNA sequencing based on KEGG pathways enrichment shows the top 10 enriched pathways in USP22‐knockdown A375 cells. (C) Gene Set Enrichment Analysis (GSEA) showing PI3K–Akt pathway and EMT signatures were enriched in control group (shCtrl) based on RNA sequencing. (D) GSEA showing PI3K–Akt pathway and EMT signatures were enriched in USP22‐high group based on Xiangya cohort. (E) The protein expression levels of Akt, p‐Akt (Ser473), mTOR, p‐mTOR (Ser2448) in USP22‐knockout (sgUSP22) and USP22‐overexpressing (USP22) A375 cells quantified by western blotting. (F and G) Transwell assay showing effect of GDC0941 (PI3Ki, 1 µM), MK2206 (Akti, 8 µM), AZD8055 (mTORi, 500 nM) on the role of USP22 silencing (sgUSP22) in A375 cell migration. Two‐way ANOVA analysis was performed in (G). Ns., not significance; ****p < 0.0001.

To further elucidate the regulatory mechanism of USP22 in melanoma EMT, RNA sequencing was conducted on USP22 knockdown and control A375 cell lines (Figure 4A). Strikingly, PI3K/Akt pathway, one of the pathways most significantly affected by USP22, was implicated in playing a crucial role (Figure 4B). Gene Set Enrichment Analysis (GSEA) further illustrated that USP22 markedly activates PI3K/Akt/mTOR pathway (Figure 4C). As expected, the EMT pathway was inactivated in the USP22 knockdown group (Figure 4C), thus supporting our aforementioned findings (Figure 3). To substantiate these results, we performed GSEA based on the Xiangya melanoma datasets. The analysis showed significant enrichment of both the PI3K/Akt/mTOR signaling pathway and the EMT pathway in the USP22 high group (Figure 4D). Notably, the PI3K/Akt pathway has been reported to regulate tumor metastasis through EMT,^30^ highlighting the possibility of USP22 in promoting melanoma metastasis and EMT via the PI3K/Akt pathway. To test this hypothesis, we further assessed the association between USP22 and PI3K/Akt/mTOR pathway following USP22 inhibition or overexpression. We found that USP22 knockout results in a decrease in the phosphorylation of Akt at Ser473 and mTOR at Ser2448, while USP22 overexpression increased phosphorylation of Akt at Ser473 and mTOR at Ser2448, with minimal effects on total Akt and mTOR levels (Figure 4E). USP22 inhibition, PI3K inhibitor (GDC‐0941), Akt inhibitor (MK‐2206), and mTOR inhibitor (AZD‐8055) strongly inhibited melanoma invasion in melanoma cells (Figure 4F,G). However, USP22 inhibition could not further enhance the inhibitory effect on melanoma invasion in the presence of PI3K inhibitor (GDC‐0941), Akt inhibitor (MK‐2206), or mTOR inhibitor (CCI‐779) (Figure 4F,G). These results suggested that USP22 potentiates melanoma metastasis and EMT through activating the PI3K/Akt/mTOR pathway.

### 2.5 USP22 activates PI3K/Akt/mTOR pathway via SIRT1/PTEN axis

**FIGURE 5:**
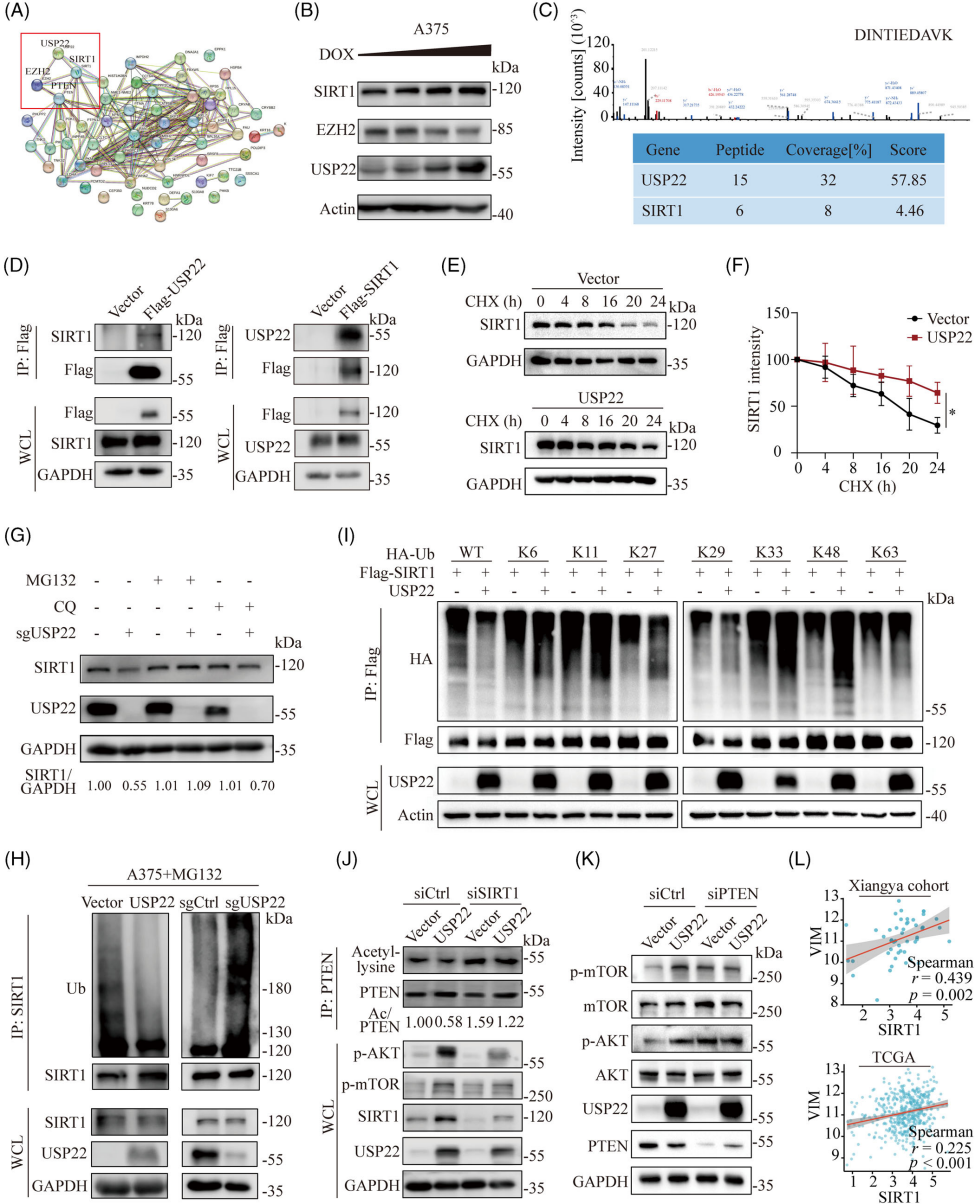
USP22 activates PI3K/Akt pathway via SIRT1/PTEN axis. (A) Protein–protein interactions (PPI) analysis containing USP22, USP22 binding proteins and proteins involved in modulating PI3K–Akt signal pathways. Proteins interacting with USP22 were identified from immunoprecipitation and mass spectrometry (IP‐MS) analysis. (B) Western blotting analysis showing SIRT1 and EZH2 protein expression in USP22‐inducible cells with doxycycline (0–1 µM, wedge). (C) The peak map of SIRT1 acquired from the mass spectrometry. (D) A375 cells were transfected with vectors encoding Flag‐tagged USP22 and Flag‐tagged SIRT1 respectively. Protein lysates then were harvest for immunoprecipitated with anti‐Flag beads, then immunoblotted with the SIRT1 and USP22. (E) Protein lysates of control and USP22‐overexpressing (USP22) A375 cells were treated with cycloheximide (CHX) (100 µg/mL) for the indicated time points, followed by immunoblotting with SIRT1. (F) Quantification of the data from (E) (*n* = 3 biologically independent samples). (G) Protein lysates of control and USP22‐knockout (sgUSP22) A375 cells were treated with DMSO, MG132 (10 µM) and CQ (20 µM) for 6 h respectively, and followed by immunoblotting with SIRT1 and USP22. (H) Protein lysates of USP22‐overexpressing (USP22) and USP22‐knockout (sgUSP22) A375 cells harvested after MG132 (10 µM) treatment (6 h) for immunoprecipitated with ubiquitin (Ub) antibody plus protein A/G beads and immunoblotted with SIRT1. (I) Immunoblot analysis of 293T cells transfected with Flag‐SIRT1, HA‐tagged ubiquitin, HA‐tagged K6‐linked ubiquitin, HA‐tagged K11‐linked ubiquitin, HA‐tagged K27‐linked ubiquitin, HA‐tagged K29‐linked ubiquitin, HA‐tagged K33‐linked ubiquitin, HA‐tagged K48‐linked ubiquitin, and HA‐tagged K63‐linked ubiquitin with or without USP22, followed by treatment with MG132 and IP with anti‐Flag beads, then detected with anti‐HA. (J) USP22‐overexpressing (USP22) and control A375 cells were transfected with SIRT1 siRNAs. Protein lysates were harvested for immunoprecipitated with PTEN antibody plus protein A/G beads and immunoblotted with acetyl‐lysine. (K) Western blot analysis of PI3K/Akt/mTOR changes in indicated control and USP22‐overexpressing (USP22) cells after transfection with PTEN siRNAs. (L) Spearman's correlation between vimentin (VIM) and SIRT1 based on data from Xiangya cohort and TCGA‐SKCM.

To further dissect the precise mechanism by which USP22 activates the PI3K/Akt signaling pathway, coimmunoprecipitation (Co‐IP) and mass spectrometry (MS) were utilized to identify the potential direct binding partners of USP22. Protein–protein interaction analysis was performed among USP22, its binding proteins and proteins involved in regulating the PI3K/Akt signaling pathway (Figure 5A). SIRT1 and EZH2 emerged as key mediators linking USP22 to PTEN, a major negative regulator of the PI3K/Akt pathway (Figure 5A). To clarify the regulatory relationships between USP22 and SIRT1 and EZH2, we treated USP22‐inducible cells with doxycycline and found USP22 could stabilize SIRT1 but not EZH2 (Figures 5B and S4A,B). Consistently, MS and Co‐IP analysis verified the interaction between USP22 and SIRT1 (Figure 5C,D). Furthermore, USP22 overexpression markedly extended the half‐life of SIRT1 by a cycloheximide assay (Figure 5E,F). The USP22 inhibition‐induced decrease in SIRT1 expression was partially reversed by treatment with the proteasome inhibitor MG132, but not the autophagy inhibitor chloroquine (Figure 5G), suggesting that USP22 may inhibit the degradation of SIRT1 through a proteasome‐dependent pathway. USP22 functions as a deubiquitinase that acts by removing ubiquitin from target proteins through direct interaction.^14^ As expected, USP22 inhibition increased, while USP22 overexpression decreased the abundance of SIRT1 ubiquitination (Figure 5H). To determine the type of ubiquitin chain cleavage by USP22 on SIRT1, we overexpressed hemagglutinin‐tagged wild‐type ubiquitin or mutated six of the seven lysines to arginine (e.g., K48), together with flag‐tagged SIRT1 in 293T cells. Notably, overexpression of USP22 effectively decreased K29‐linked ubiquitination of SIRT1 (Figure 5I). These results suggested that USP22 stabilizes SIRT1 by reducing its ubiquitination, thereby inhibiting its proteasome‐mediated degradation.

SIRT1 has been reported to deacetylate PTEN and activate the PI3K/Akt/mTOR pathway.^31^ Deacetylated PTEN is well known to activate PI3K/AKT/mTOR pathway through promoting its phosphatase activity.^32^, ^33^, ^34^ To determine whether USP22 activates the PI3K/Akt/mTOR pathway via SIRT1‐mediated PTEN deacetylation, we used siRNA to silence the expression of SIRT1 in USP22‐overexpressing melanoma cells. We found that USP22‐mediated SIRT1 upregulation inhibits the acetylation of PTEN and activates the PI3K/Akt/mTOR pathway, which is partially abolished by SIRT1 knockdown (Figure 5J). Moreover, PTEN inhibition could also reverse the USP22‐mediated activation of the PI3K/Akt/mTOR pathway (Figure 5K). Notably, SIRT1 expression is positively correlated with the EMT marker vimentin using Xiangya melanoma datasets and TCGA datasets (Figure 5L). These findings suggested that USP22 activates the PI3K/Akt/mTOR pathway and induces melanoma EMT via the SIRT1/PTEN axis.

### 2.6 Drug screening identifies topotecan as a USP22‐targeting molecule to suppress melanoma metastasis

**FIGURE 6:**
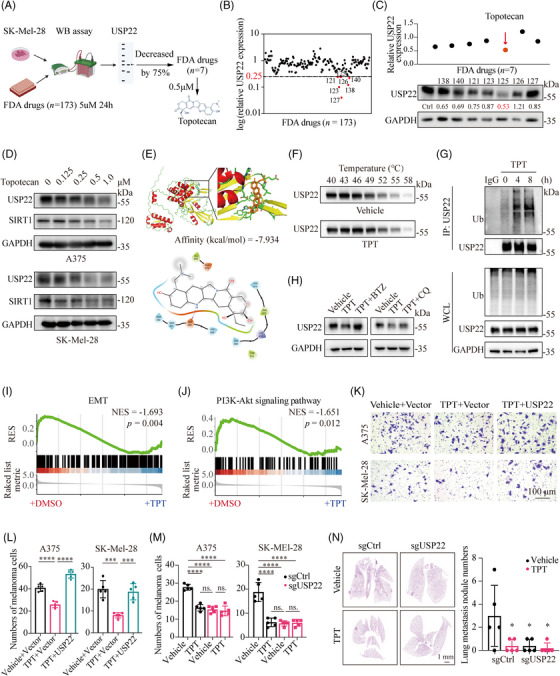
Identification of USP22‐targeting molecule to suppress melanoma metastasis. (A) Schematic view of western blotting‐based drug screening. (B) Screening with US FDA‐approved drug library (5 µM) for 24 h identified seven out of 173 compounds that decreased USP22 expression by 75%. (C) Screening with US FDA‐approved drug library (0.5 µM) for 24 h identified topotecan as the most efficient USP22‐targeting molecule (*n* = 1). (D) Western blotting showing the protein levels of USP22, SIRT1 in A375 and SK‐Mel‐28 cells treated with topotecan for 24 h. (E) Prediction of the interaction between topotecan and USP22 protein. (F) The cellular thermal shift assay (CETSA) of USP22 proteins for A375 cells treated with DMSO (Vehicle) or topotecan (TPT, 0.5 µM). (G) An IP assay was performed to verify ubiquitin (Ub) modification of USP22 in A375 cells after treatment with topotecan (TPT, 0.5 µM) for 4–8 h and MG132. (H) Western blot analysis of the USP22 expression in A375 cells following treatment with topotecan (TPT, 0.5 µM) in the absence or presence of bortezomib (BTZ; 0.2 µM) or chloroquine (CQ; 20 µM) for 24 h. (I and J) GSEA showing less enrichment of EMT (I) and PI3K–Akt pathway (J) in SK‐Mel‐28 cells treated with topotecan (TPT) for 24 h than cells treated with DMSO. (K–M) Transwell assay showing effect of topotecan (TPT, 0.5 µM) on the migration of USP22‐overexpressing (USP22) (K and L) or USP22‐knockout (sgUSP22) (M) A375 and SK‐Mel‐28 cells. (N) Representative H&E images of lung metastasis of mice in the indicated groups. Two‐way ANOVA analysis was performed in (L–N). *p < 0.05, ***p < 0.001, ****p < 0.0001.

Currently, the clinical applicability of USP22 inhibitors is limited due to their inefficiency and lack of specificity. To identify potential USP22‐targeting molecules with clinical relevance, we conducted a screening of 173 antitumor drugs obtained from the US FDA‐approved drug library using western blotting assay in SK‐Mel‐28 melanoma cells (Figures 6A and S5).^35^ Seven compounds were identified to suppress USP22 expression by 75% under a concentration of 5 µM (Figure 6B). Subsequently, we conducted screening with a lower concentration (0.5 µM) and topotecan emerged as the most effective drug to downregulate USP22 expression (Figure 6C). To further verify our findings, we treated with A375 and SK‐Mel‐28 cell lines with varying dosages of topotecan, and the results showed that topotecan inhibits USP22 expression as well as its downstream SIRT1 expression in a dose‐dependent manner (Figure 6D). Next, we investigated how topotecan downregulates USP22 expression. Molecular docking assays indicated that topotecan could potentially interact with USP22 (Figure 6E). Within the binding site, topotecan establishes a hydrogen bond with Asp262 and hydrophobic interactions with Gln260, Gln261, His264, His408, Lys410, Phe412, His414, Leu475, Glu476, and Tyr513 (Figure 6E). Cellular thermal shift assay (CETSA) confirmed that topotecan increases the thermal stability of the USP22 protein in melanoma cells, indicating that topotecan directly interacts with USP22 (Figures 6F and S6A). Treatment with topotecan had no significant effect on USP22 mRNA levels (Figure S6B,C), but facilitated the ubiquitination of USP22 (Figure 6G). Bortezomib, a proteasome inhibitor, but not chloroquine, an autophagy inhibitor, prevented topotecan‐induced USP22 protein degradation (Figure 6H). These findings indicate that topotecan binds to USP22 and promotes its degradation by the ubiquitin‐proteasome pathway.

Notably, RNA sequencing revealed a downregulated enrichment in EMT and PI3K/Akt pathways under topotecan treatment, as demonstrated by GSEA analysis (Figure 6I,J). Consistently, topotecan treatment decreased SIRT1 expression, phosphorylation of Akt at Ser473 and mTOR at Ser2448, as well as EMT activity, but had minimal effects in USP22‐deficient melanoma cells (Figure S6D). Moreover, the attenuated migration capacity induced by topotecan was significantly abolished by USP22 overexpression (Figure 6K,L). Topotecan treatment‐induced downregulation of cell migration was not further decreased under USP22 deficiency (Figures 6M and S6E). In vivo assays further suggested that topotecan has an equivalent effect to genetic silencing of USP22 in inhibiting melanoma lung metastasis, but topotecan could not further decrease lung metastasis in the absence of USP22 (Figure 6N). Taken together, these results suggested that topotecan suppresses melanoma metastasis by specifically targeting USP22.

### 2.7 USP22 expression controls melanoma vulnerability to ferroptosis

**FIGURE 7:**
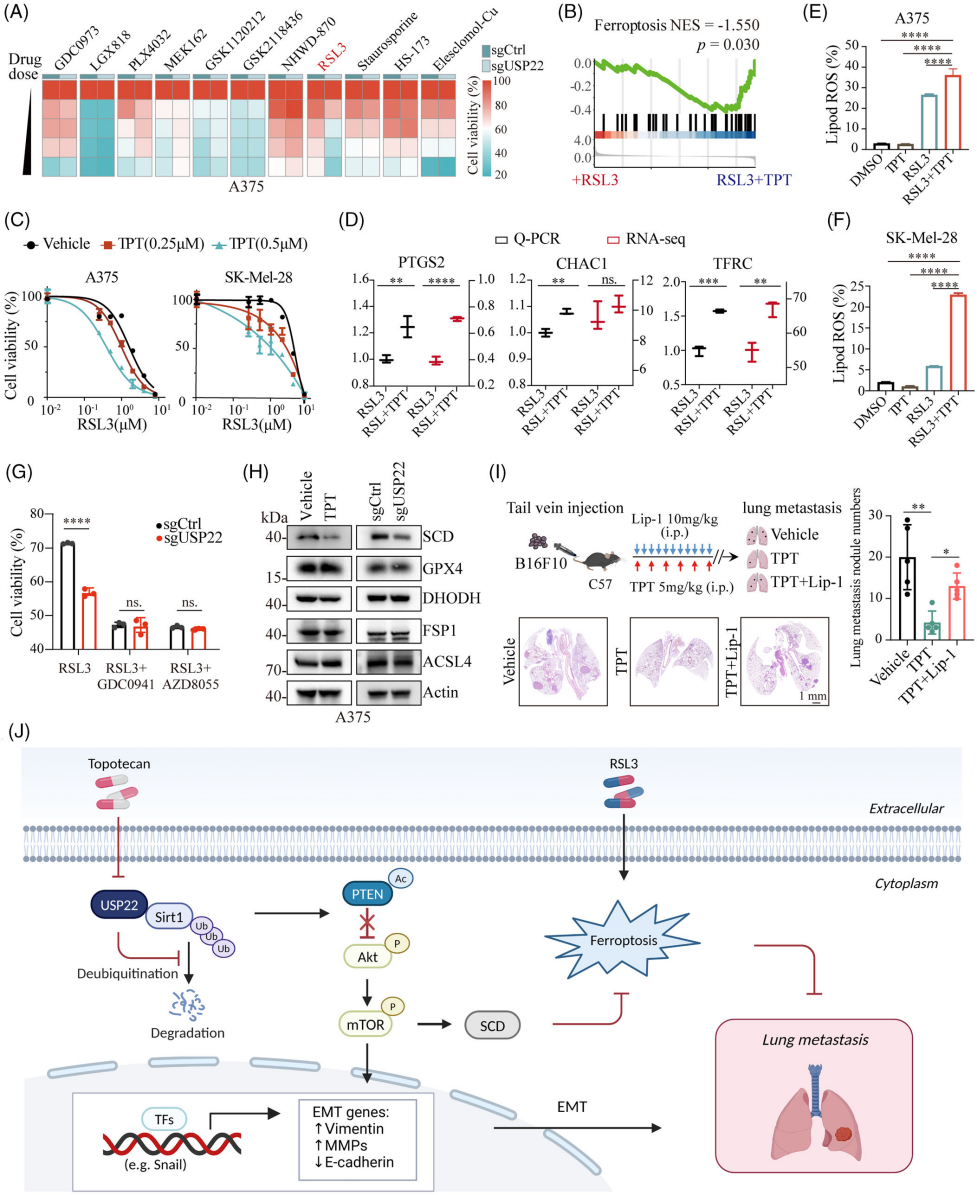
Inhibition of USP22 sensitizes melanoma to ferroptosis. (A) Heatmap showing cell viability of USP22‐knockout (sgUSP22) and control (sgCtrl) A375 cells treated with BRAF inhibitors (LGX818, GSK2118436, PLX4032), MEK inhibitors (GDC0973, GSK1120212, MEK162), and BET inhibitor (NHWD‐870), ferroptosis inducer (RSL3), apoptosis inducer (staurosporine), necroptosis inducer (HS‐173), and cuproptosis inducer (Elesclomol‐Cu). (B) GSEA showing enrichment of ferroptosis in RSL3‐treated SK‐Mel‐28 cells after topotecan (TPT) exposure for 24 h. (C) Cell viability of A375 and SK‐Mel‐28 cells treated with RSL3 or topotecan (TPT) either alone or in combination for 24 h. (D) Real‐time PCR and RNA sequencing showing the mRNA expression of ferroptosis marker including PTGS2, CHAC1, and TFRC in SK‐Mel‐28 cells treated with RSL3 alone or in combination with topotecan (TPT). (E and F) The levels of lipid ROS detected by C11‐BODIPY 581/591 in A375 (E) and SK‐Mel‐28 cells (F) exposed to RSL3 (2.5 µM), TPT (0.5 µM) or the combination. (G) Cell viability of USP22‐knockout (sgUSP22) and control (sgCtrl) A375 cells treated with RSL3 (2.5 µM) alone or plus GDC0941 (PI3Ki, 1 µM) or AZD8055 (mTORi, 500 nM). (H) Western blotting indicated the levels of ferroptosis‐associated proteins (SCD, GPX4, DHODH, FSP1, and ACSL4) in A375 cells after treatment with topotecan (TPT, 0.5 µM) or USP22‐knockout (sgUSP22). (I) Schematic view and representative H&E images of lung metastasis of mice after tail vein injection of B16F10 cells in the indicated groups. (J) Graphical summary showing that targeting USP22 by topotecan suppresses melanoma metastasis via inhibiting EMT and sensitizes ferroptosis through SIRT1/PTEN/PI3K axis. One‐way ANOVA analysis was performed in (D–G, I). ns., no significance, *p < 0.05, **p < 0.01, ***p < 0.001, ****p < 0.0001.

Inducing cell death serves as a crucial mechanism for the elimination of melanoma cells.^20^, ^36^, ^37^ To explore the effect of USP22 on current melanoma treatment and cell death inducers, we screened the vulnerability of USP22‐deficient melanoma cells to a series of antimelanoma drugs, including BRAF/MEK inhibitors, NHWD‐870 (a novel oral BET inhibitor developed by our team),^38^, ^39^, ^40^ and cell death inducers, such as inducers of apoptosis (staurosporine), necroptosis (HS‐173), ferroptosis (RSL3), and cuproptosis (elesclomol‐Cu) (Figures 7A and S7A). We found that USP22 deficiency only enhances the vulnerability of melanoma to ferroptosis inducer RSL3 and has no sensitizing effect on other antimelanoma drugs or cell death inducers (Figures 7A and S7A), suggesting that USP22 has a specific sensitization effect on melanoma ferroptosis. The sensitized effect of USP22 on RSL3‐induced ferroptosis could be completely rescued by N‐acetylcystiene, an aminothiol and synthetic precursor of intracellular cysteine and GSH, further supporting our findings (Figure S7B). In line with this finding, we investigated the transcriptomic data from A375 cells treated with RSL3 and topotecan, or RSL3 alone. GSEA demonstrated that the pathway associated with ferroptosis was significantly enriched in the group of RSL3 plus topotecan (Figure 7B). Topotecan sensitized melanoma cells to RSL3‐induced ferroptosis in a dose‐dependent manner, with left shift of dose‐response curves (Figure 7C). The mRNA levels of PTGS2, CHAC1, and TFRC, the biomarkers of ferroptosis, are also highly expressed in the combination group by RNA‐seq and RT‐PCR analyses (Figure 7D). Consistently, the lipid peroxidation levels, the hallmark of ferroptosis, are also increased in the combination group (Figure 7E,F). These results suggested that UPS22 inhibition sensitizes melanoma cells to ferroptosis.

Previously, we reported that inhibition of PI3K/Akt/mTOR pathway‐mediated SCD expression could sensitize melanoma to ferroptosis.^41^ We thus examined whether USP22 silencing sensitizes RSL3‐induced ferroptosis through the PI3K–Akt–mTOR pathway. We found that USP22 knockout failed to further sensitize RSL3‐induced ferroptosis in the presence of PI3K inhibitor GDC0941 or mTOR inhibitor AZD8055 (Figure 7G). Moreover, pharmacological or genetic inhibition of USP22 significantly downregulated SCD expression, but not other ferroptosis‐related proteins, including ACSL4, GPX4, FSP1, and DHODH (Figures 7H and S7C). These results demonstrated that USP22 controls melanoma vulnerability to ferroptosis at least partly through targeting the PI3K/Akt/mTOR pathway‐mediated SCD expression. Metastasizing melanoma cells in blood and lymph are protected from ferroptosis to promote the formation of metastatic tumors.^22^ To further collaborate this finding, we performed the tail vein experimental metastasis assay. Treatment with ferroptosis inhibitor liproxstatin‐1 significantly alleviated topotecan‐mediated inhibition of melanoma lung metastasis (Figure 7I), indicating that topotecan inhibits the metastatic potential of melanoma at least by promoting ferroptosis. Taken together, these results suggested that inhibition of USP22 enhances ferroptosis sensitivity by downregulating SCD, thereby contributing to the reduction of melanoma metastasis (Figure 7J).

## 3 DISCUSSION

Accumulating evidence has shown that malfunction of USPs is linked to tumor metastasis, implicating that targeting USPs could provide new strategies for the treatment of advanced melanoma.^9^, ^42^ In this study, by analyzing the profile survival of 52 USPs, we identified USP22 as a predictor for poor prognosis in melanoma. USP22 expression is highly upregulated in melanoma and positively associated with the pathological stage of melanoma, suggesting the potential of USP22 as a diagnostic and prognostic biomarker for melanoma. Previous studies have shown USP22 could regulate melanoma cell proliferation^18^; however, upon examining more melanoma cell lines, we found a slight, not significant role of USP22 on melanoma cell proliferation and melanoma growth in vivo. Conversely, we have substantiated a remarkable role of USP22 in promoting melanoma migration, invasion, and lung metastasis.

USP22 is reported to exert as a metastasis promoter through various mechanisms.^14^ However, our current understanding of the role of USP22 in melanoma metastasis remains largely unexplored. Herein, we conducted RNA sequencing and found that USP22 promotes melanoma metastasis through the PI3K/Akt pathway. Inhibition of the PI3K/Akt pathway could rescue the effects of USP22 on cell migration. Considering that USP22 typically functions through interactions with different proteins and regulates protein stability via deubiquitination, we utilized MS to investigate the binding partners of USP22 and revealed that USP22 interacts with SIRT1 and stabilizes SIRT1 expression through its deubiquitinase activity. It has been reported that SIRT1 contributes to melanoma progression.^43^ We took a further step to elucidate the role of USP22/SIRT1 interaction in melanoma metastasis. Specifically, SIRT1 serves as a unique bridge connecting USP22 and PI3K/Akt association. SIRT1 can activate the PI3K/Akt pathway by deacetylating PTEN. PTEN acetylation is important for its activation, and PTEN is a key suppressor of the PI3K/Akt pathway.^31^, ^44^ In agreement with previous results, we found that interference of SIRT1 increases the acetylation of PTEN and impairs PI3K/Akt activation.

Our study highlights the crucial role of USP22 in melanoma metastasis, presenting it as an attractive therapeutic target. Several USP22 inhibitors have been developed, including the first macrocycle USP22 inhibitor hD1 peptide,^45^ the first small USP22‐targeting agent USP22i‐S02,^46^ and the most recent USP22 inhibitors Rottlerin and Morusin.^47^ All of these potent USP22 inhibitors have demonstrated respectable antitumor activities in preclinical research, underscoring the clinical value of USP22 in cancer treatment. However, the clinical development of USP inhibitors is still evolving, and to date, no USP22 small molecule inhibitor has entered clinical trials. To identify potential USP22‐targeting molecular inhibitors applying for clinic, we performed a screening of the US FDA‐approved drug library and discovered topotecan as a USP22‐targeting drug. We found that topotecan interacts with USP22 and facilitates its degradation through a proteasome‐dependent mechanism. As a possibility, topotecan may function as a “molecular glue” degrader,^48^ exhibiting binding affinity towards USP22 and its E3 ubiquitin ligase. Topotecan is a chemotherapeutic drug widely used in the treatment of cervical cancer, small cell lung cancer, ovarian cancer, and breast cancer.^49^ Of note, topotecan has been reported to exhibit antitumor efficiency by suppressing the PI3K/Akt pathway.^50^ However, the mechanism by which topotecan regulates the PI3K/Akt pathway remains poorly understood. In this context, our data clearly showed that topotecan impairs the PI3K/Akt pathway and EMT through the USP22/SIRT1/PTEN axis in melanoma. Pharmacological inhibition of USP22 by topotecan exerts a similar effect to USP22 knockout in inhibiting melanoma metastasis both in vivo and in vitro. These findings exhibit the crucial therapeutic effects of topotecan on advanced melanoma.

Ferroptosis, an iron‐dependent form of regulated cell death, is characterized by the accumulation of lipid peroxidation and iron, which holds promise for melanoma treatment.^51^ Previous studies have identified that ferroptosis escape might mediate melanoma cell metastasis.^22^ For example, melanoma cells in lymph experienced decreased oxidative stress and ferroptosis, resulting in the formation of more metastases than melanoma cells in blood.^22^ Li et al.^52^ further found that pharmacological targeting of 7‐DHC, a key regulator of ferroptosis, induced ferroptosis in B16F10 melanoma cells, thereby impairing metastasis. Prompted by these findings, enhancing ferroptosis sensitivity might be a promising way to suppress melanoma progression. Based on our results, the ferroptosis inhibitor liproxstatin‐1 partially rescued the inhibitory effect of topotecan on metastasis, indicating that pharmacological inhibition of USP22 could partially suppress melanoma metastasis by enhancing ferroptosis sensitivities.

Previously, Yi et al.^53^ revealed that activating mutations in PI3K or loss of PTEN mediate the ferroptosis resistance of cancer cells. We have previously reported that inhibition of the PI3K/Akt pathway could sensitize melanoma to ferroptosis via downregulating SCD.^41^ Although the role of the PI3K/Akt/mTOR pathway in regulating melanoma ferroptosis has been elucidated, its upstream signals remain enigmatic. Correspondingly, our data showed that pharmacological and genetic inhibition of USP22 could sensitize RSL3‐induced ferroptosis through inhibition of PI3K/Akt/mTOR pathway‐mediated SCD expression. Together, in comparison with other preclinical metastatic targets, our study has identified topotecan as a clinically USP22‐targeting drug capable of potently inhibiting melanoma metastasis. This discovery paves the way for a potential therapeutic application combining topotecan with ferroptosis induction in the treatment of advanced melanoma.

## 4 CONCLUSIONS

In summary, our study not only illustrates the mechanism by which USP22 controls melanoma metastasis and ferroptosis through the SIRT1/PTEN/PI3K axis but also provides a basis for the treatment of melanoma metastasis by targeting USP22.

## 5 MATERIALS AND METHODS

### 5.1 Cell culture

A375, SK‐Mel‐28, B16F10, A2058, and PIG1 cells were obtained from American Type Culture Collection. All cells were cultured in DMEM supplemented with 100 IU/mL penicillin/streptomycin and 10% FBS, and authenticated with STR profiling.

### 5.2 Reagents and antibodies

Cycloheximide (HY‐12320), MG132 (HY‐13259), GDC0941 (HY‐50094), AZD8055 (HY‐10422), MK2066 (HY‐108232), staurosporine (HY‐15141), HS‐173 (HY‐15868), and elesclomol (HY‐12040) were purchased from MedChemExpress. US FDA‐approved antitumor drugs, topotecan (S9321), GSK2118436 (S2807), GDC0973 (S8041), LGX818 (S7108), PLX4032 (S1267), GSK1120212 (S2673), doxycycline (S5159), and chloroquine (S6999), were purchased from Selleck Chemicals. NHWD‐870 was a gift from Ningbo Wenda Pharma, China.

Proteins were incubated with antibodies as follows: USP22 (ab195289; Abcam), p‐Akt (#4060; Cell Signaling Technology), Akt (4691S; Cell Signaling Technology), p‐mTOR (#2971; Cell Signaling Technology), mTOR (#2983; Cell Signaling Technology), PTEN (#9188; Cell Signaling Technology), acetyl‐lysine (#9441; Cell Signaling Technology), ubiquitin (#3936; Cell Signaling Technology), GPX4 (#52455; Cell Signaling Technology), SIRT1 (#30086; ProMab), EZH2 (#P01006; ProMab), IgG (Beyotime Biotechnology), vimentin (sc‐6260; Santa Cruz Biotechnology), CDH1 (sc‐8426; Santa Cruz Biotechnology), SNAI1 (sc‐271977), FSP1 (20886‐1‐AP; Proteintech), DHODH (#26381; Cell Signaling Technology), ACSL4 (sc‐365230; Santa Cruz Biotechnology), SCD (sc‐81776; Santa Cruz Biotechnology), GAPDH (sc‐47724; Santa Cruz Biotechnology), and Actin (sc‐8432; Santa Cruz Biotechnology).

### 5.3 Quantitative real‐time PCR

RNA was isolated and then used for cDNA synthesis. The gene expression level was relative to the level of GAPDH. Primers for real‐time PCR were summarized in Table S1.

### 5.4 Western blotting and immunoprecipitation

For immunoprecipitation, cells were lysed with NP40 buffer (Beyotime Biotechnology), incubated with antibodies along with Protein A/G beads or anti‐Flag agarose beads (Sigma) overnight, and washed with NP‐40 buffer. Precipitated proteins were boiled with 2×SDS Loading Buffer and then subjected to SDS‐PAGE electrophoresis. Western blotting analysis was conducted as described previously.^35^

### 5.5 RNA interference and lentiviral transduction

For RNA interference, siRNAs were obtained from Obio Technology (Shanghai, China). USP22 vector was obtained from Fenghui Biotechnology (Changsha, China). SIRT1 vector was purchased from Youze Biotechnology (Changsha, China). Transfection with siRNA, sgRNA, and vectors were conducted with TurboFect. Package plasmid was necessary for the construction of stable cells. After 2 days, target cells were transduced with the virus suspensions, followed by selection in the presence of 2 µg/mL puromycin for 1−3 days. The sequences of the siRNAs, shRNA, and sgRNA were listed in Table S2.

### 5.6 Immunohistochemistry

Melanoma patient tissue microarrays (DC‐Mel11015) for IHC staining were obtained from Avilabio (Xian, China) and detailed information of tissue array was listed in Table S3. The tissue samples were stained with USP22 antibodies according to methods previously described.^35^ The staining intensity of USP22 (ranged from 0 to 3) and the percentage of tumor cell area (ranged from 0 to 100%) were used to generate a *H*‐score [*H*‐score = ∑(pi × *i*) = (percentage of weak intensity area × 1) + (percentage of moderate intensity area × 2) + (percentage of strong intensity area × 3)], ranged from 0 to 300.

### 5.7 RNA sequencing and MS analysis

For RNA sequencing, total RNA was extracted from USP22 knockdown and control A375 cells for RNA sequencing analysis. Nuclei were sequenced using the Novaseq6000 PE150 platform with manufacturer's instructions.^54^ Differential expressed genes were defined as those with a | log2‐fold‐change | > 1 and *p* value < 0.05. The data of RNA sequencing with USP22 knockdown and topotecan treatment were listed in Tables S4 and S5, respectively. The data of RNA sequencing with topotecan and RSL3 combination versus RSL3 treatment were listed in Table S6.

For MS analysis, Flag‐tagged USP22 was overexpressed in A375 cells. The cell lysates were immunoprecipitated using anti‐Flag beads (Sigma) and used for MS (Thermo Q ExactiveTM HF‐X). The MS raw data were analyzed and aligned using Proteome Discoverer 2.2 software. The parameters were set as follows: a mass tolerance of 10 ppm for precursor ions and a mass tolerance of 0.02 Da for fragment ions. Two missed cleavage sites are allowed. The PD2.2 software further filtered to enhance the quality of analysis results: only credible spectral peptides and proteins were retained. FDR verification was conducted by removing peptides and proteins with an FDR exceeding 1%. Library construction and data processing for both RNA‐seq and MS were performed by Nocogene, China.

### 5.8 Migration and invasion assay

For 3D Matrigel drop invasion assay, a total of 5 × 10^4^ A375, SK‐Mel‐28, B16F10 cells were suspended in 10 µL Matrigel and pipetted into a 24‐well plate as a droplet. The Matrigel drop was incubated at 37°C for 15 min, and then added media containing 10% FBS. On Day 6, the distance of cell migration away from the edge of drop was measured as radial migration.

For transwell assay, cells suspended in serum‐free medium were placed onto 6.5 mm × 8.0 µm transwell chamber inserts precoated with (invasion assay) or without (migration assay) matrigel (1:7 mix with serum‐free medium) (Corning). Medium containing 30% FBS was added in the lower chamber. After 12−20 h, the migrated or invaded cells were fixed and then stained with crystal violet. Finally, the migrated cells were examined by microscope.

### 5.9 Drug screening

A commercial US FDA‐approved drug library was purchased from Selleck Chemicals. A total of 173 antitumor drugs were utilized to identify potential USP22‐targeting molecule. 1 × 10^4^ SK‐Mel‐28 cells were treated with US FDA‐approved drugs under a concentration of 5 µM for 24 h in 96‐well plate. Then western blotting analysis was conducted with USP22 antibody incubated. Images were required and top seven compounds were identified to suppress USP22 expression by 75%. Subsequently, similar screening with a lower concentration (0.5 µM) were performed with western blotting assay.

### 5.10 Lipid peroxidation assay

Cells were seeded onto six‐well plates, incubated with the indicated compounds, and washed by PBS. After collecting, cells were stained with 2.5 µM C11‐BODIPY 581/591 dye (Thermo Fisher Scientific; D3861) at 37°C for 20 min and washed with PBS to remove excess dye and resuspended with 300 µL of PBS. Lipid ROS levels were ultimately tested by flow cytometry and analyzed by FlowJo software (version 10.8.2).

### 5.11 Animal study

All animal experiment protocols and procedures were approved by the Ethical Review of the Animal Care and Use Committee of Central South University (ethics approval number: 2023‐S195). SPF grade female BALB/c nude and C57BL/6 mice were obtained, bred, and housed in the Animal Facility of Central South University.

In lung metastasis model, 1 × 10^6^ A375 and 2 × 10^5^ B16F10 cells suspended in 100 µL PBS were injected into the tail vein of nude and C57BL/6 mice, respectively. For the therapy experiments, mice received intraperitoneal injection of 5 mg/kg topotecan (Selleck, Shanghai, China) or 2% DMSO every 3 days from day 4. For ferroptosis‐associated experiments, 10 mg/kg liproxstatin‐1 (Selleck, Shanghai, China) was administered intraperitoneally to mice every day. Liproxstatin‐1 was dissolved in 2% DMSO plus 40% PEG300 plus 2% Tween. The administration route and dose of topotecan were same as above. Topotecan was dissolved in 2% DMSO plus 40% PEG300 plus 5% Tween. The body weight of all mice was monitored every 3 days. Animals were sacrificed around 7 weeks or 25 days after inoculation, and their lung tissues were collected. Metastasis tumor nodules were then examined using H&E staining.

### 5.12 Clinical data

All clinical specimen were collected in compliance with the informed consent policy and from Xiangya Hospital. All clinical specimen's data in this study were previously described.^55^

### 5.13 Fit docking assay

The protein structure data of USP22 were retrieved from UniProt, predicted using the AlphaFold method (ID: AF‐Q9UPT9‐F1), and subsequently optimized using the Protein Preparation Workflow in Maestro (Version 13.7.125). The protein‐binding pockets were identified using SiteMap in Maestro. The ligand's structure was generated using LigPrep in Maestro, and its optimization was performed by minimizing potential energy using default parameters. The Maestro's binding site detection was utilized to generate site maps, which identified the putative binding sites of the protein. The center of each grid box was positioned at the center of a binding site, and the Glide extra precision (XP) docking function was applied. The induced fit docking method was employed to accurately predict the ligand‐binding modes and the associated structural changes in the receptor.

### 5.14 Cellular thermal shift assay

Cells were incubated with 0.5 µM topotecan or vehicle for 3 h, collected, centrifuged, and aliquoted into tubes. The samples were heated at different temperatures (40−58°C, in 3°C increments) for 3 min, then cooled to room temperature, and lysed in liquid nitrogen by three freeze‐thaw cycles. Finally, the samples were centrifuged at 4°C, and the soluble fraction was diluted with 5× SDS loading buffer for further analysis.

### 5.15 Statistical analyses

The dataset GSE15605 and GSE46517 were downloaded from GEO database. For RNA‐seq data, GSVA and GSEA were conducted by R software (3.6.3). Data in this study were analyzed with GraphPad Prism 8 and R statistic software. Student's t‐test was performed to compare two groups, while ANOVA analysis was used for comparisons between multiple groups. Statistical significance performed in this paper was as follow: ns., not significance; *p < 0.05; **p < 0.01; ***p < 0.001; ****p < 0.0001.

## AUTHOR CONTRIBUTIONS

H. S., Y. M., and L. Y. carried out the experiments and analyzed the data. G. D., F. Z., and X. C. supervised the project and designed experiments. H. S. and Y. M. wrote the manuscript. Y.‐H. L. helped with animal experiments. S. D., Y.‐Y. L., Q. Z., Y.‐H. L., Y. D., Y. S., X. W., and X. L. helped edit and revise the manuscript. All authors had access to the study data and reviewed and approved the final manuscript.

## CONFLICT OF INTEREST STATEMENT

The authors have no relevant financial or nonfinancial interests to disclose.

## ETHICS STATEMENT

The melanoma tissue array with normal skin tissue for IHC staining was purchased from Avilabio. The clinical specimen for data analysis were collected in compliance with the informed consent policy for research use and from Xiangya Hospital (ethics approval number: 202308636). All animal experiments were approved by the Animal Ethics Committee of the Central South University (ethics approval number: 2023‐S195).

## CONSENT TO PUBLISH

Not applicable.

## Supporting information

Supporting Information

Supporting Information

Supporting Information

Supporting Information

## Data Availability

Data related to this paper may be requested from the corresponding author. The RNA sequencing data of USP22 knockdown are openly available in the Gene Expression Omnibus (GSE208061).
