# Supplementary material for: Ubiquitin‐specific protease 22 controls melanoma metastasis and vulnerability to ferroptosis through targeting SIRT1/PTEN/PI3K signaling
Source: MedComm (2020). 2024 Aug 12;5(8):e684. doi: 10.1002/mco2.684 (PMC11318338; doi:10.1002/mco2.684)
Supplement: Supplementary file 1 — Supporting Information [file MCO2-5-e684-s004.docx]

Supplementary Materials for

**Ubiquitin-specific protease 22 controls melanoma metastasis and vulnerability to ferroptosis through targeting SIRT1/PTEN/PI3K signaling**

Huiyan Sun^1-6#^, Yu Meng^1-5#^, Lei Yao^7#^, Songtao Du^8^, Yayun Li^9^, Qian Zhou^1-5^, Yihuang Liu^1-5^, Yating Dian^1-5^, Yuming Sun^10^, Xiaomin Wang^11^, Xiao-wei Liang^1-5^, Guangtong Deng^1-5*^, Xiang Chen^1-5*^, Furong Zeng^12*^

Correspondence to: zengflorachn@hotmail.com;

**This file includes:**

Figures. S1 to S7

Tables. S1 to S3

**
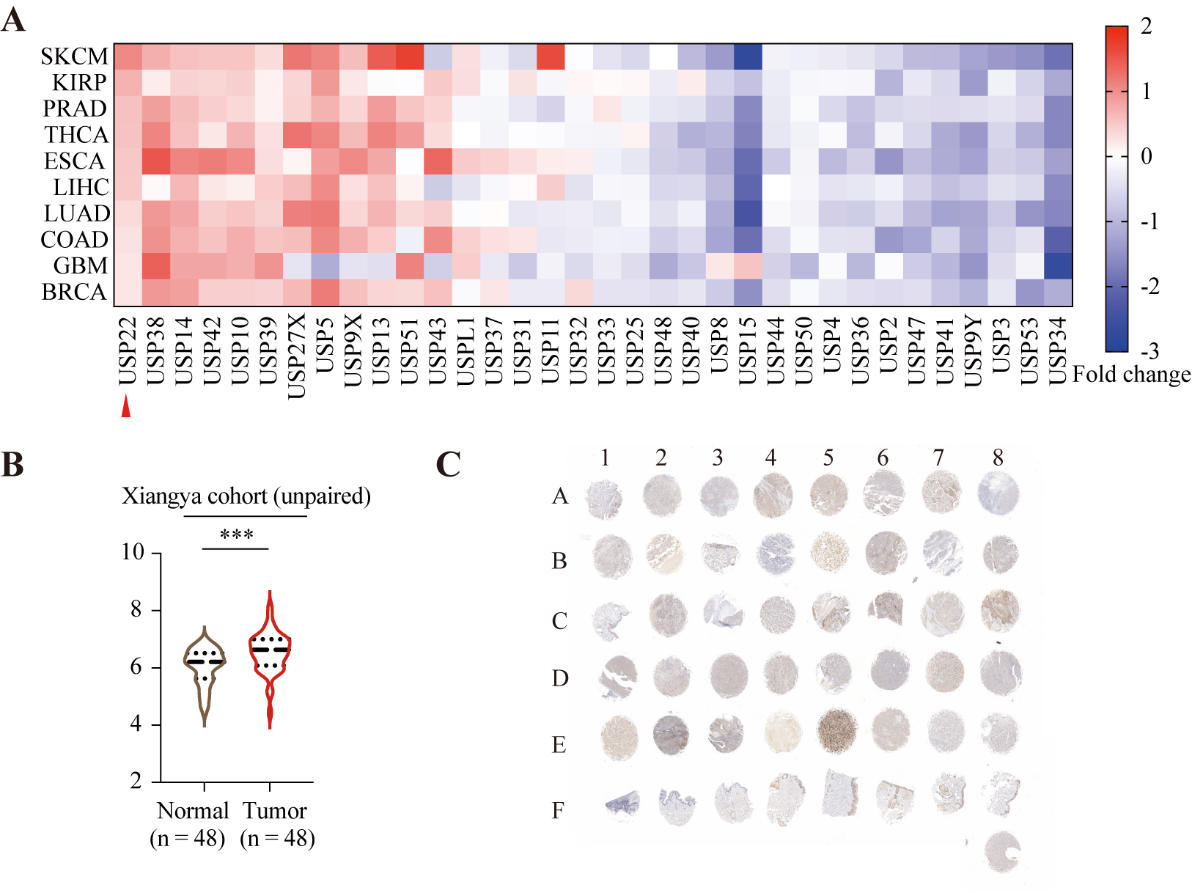
**

**Fig S1. USP22 is overexpressed in human melanoma.**

**(A)** Ubiquitin-specific proteases (USPs) differential expression analysis between tumor and normal tissues based on TCGA and GTEx databases. **(B)** USP22 mRNA expression levels in human melanoma data based on Xiangya cohort. **(C)** Immunohistochemistry staining of USP22 expression in a melanoma tissue array. Two‐tailed unpaired Student's t‐test was performed in (**B**). ***p<0.001

**
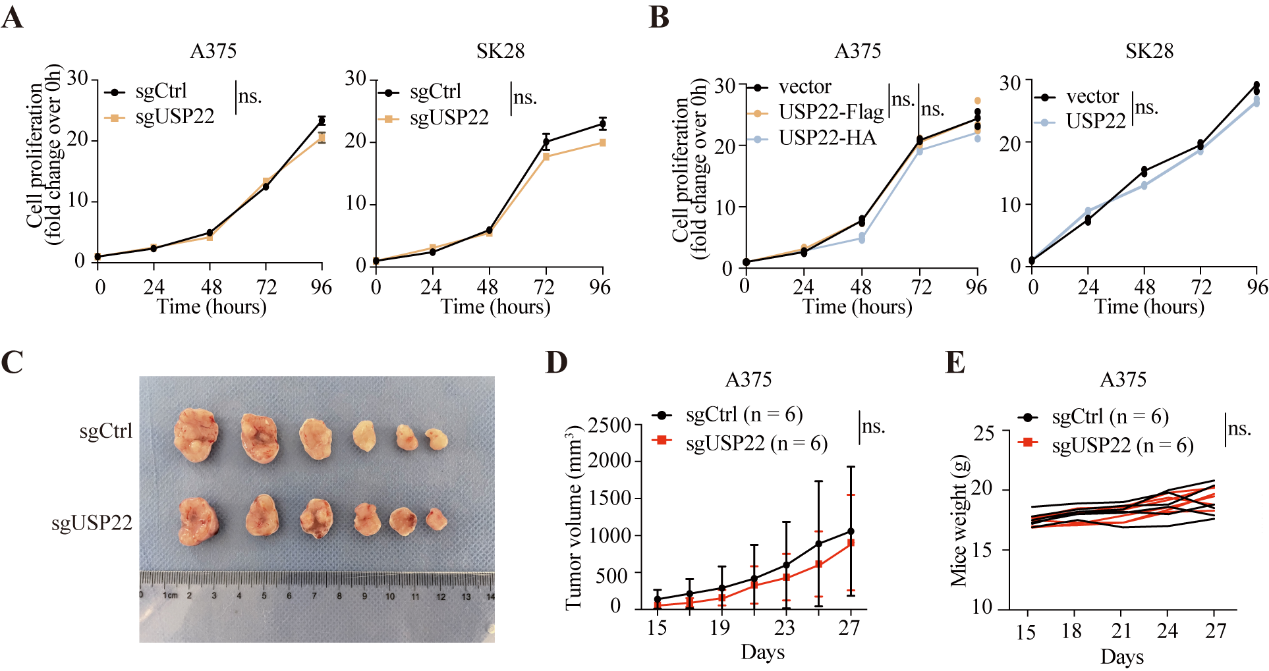
**

**Fig S2. USP22 loss have little effects on melanoma proliferation in vitro and vivo. (A-B)** Cell proliferative capacity measured by cell counting kit‐8 assay. **(C)** Images of tumors from mice xenografted with USP22-knockout (sgUSP22) and control (sgCtrl) A375 cells (n = 6 per group). **(D-E)** Tumor volume and body weight of mice in the indicated groups. Two‐way ANOVA analysis was performed in (**A-B, D-E**). Ns, no significance.

**
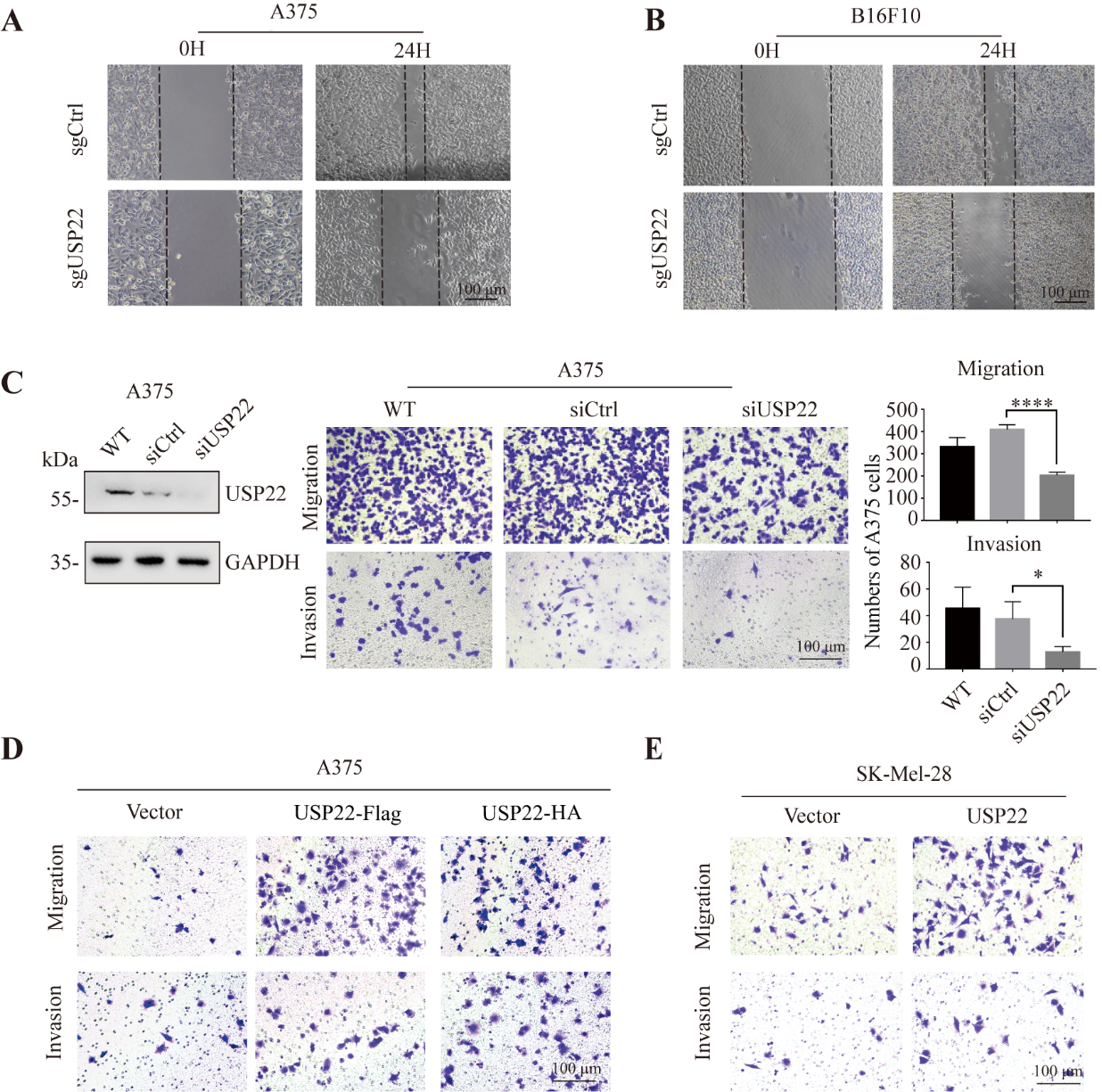
**

**Fig S3. USP22 promotes melanoma cell migration and invasion.**

**(A-B)** Wound healing assay identifying the migratory capacity after USP22-knockout (sgUSP22) in A375 (**A**) or B16F10 (**B**) cells. **(C)** Western blotting quantifying the efficiency of USP22 silencing by siRNA. Transwell assay quantifying the migration and invasive capacity after USP22 silencing with siRNA in A375 cells. **(D-E)** Transwell assay quantifying the migration and invasive capacity after USP22 overexpression in A375 (**D**) or SK-Mel-28 (**E**) cells. Migrated (without extracellular matrix) and invaded (with extracellular matrix) cells were determined for 12-20h. Five random areas were selected. One‐way ANOVA analysis was performed in (**C**). *p<0.05, ****p<0.0001.

**
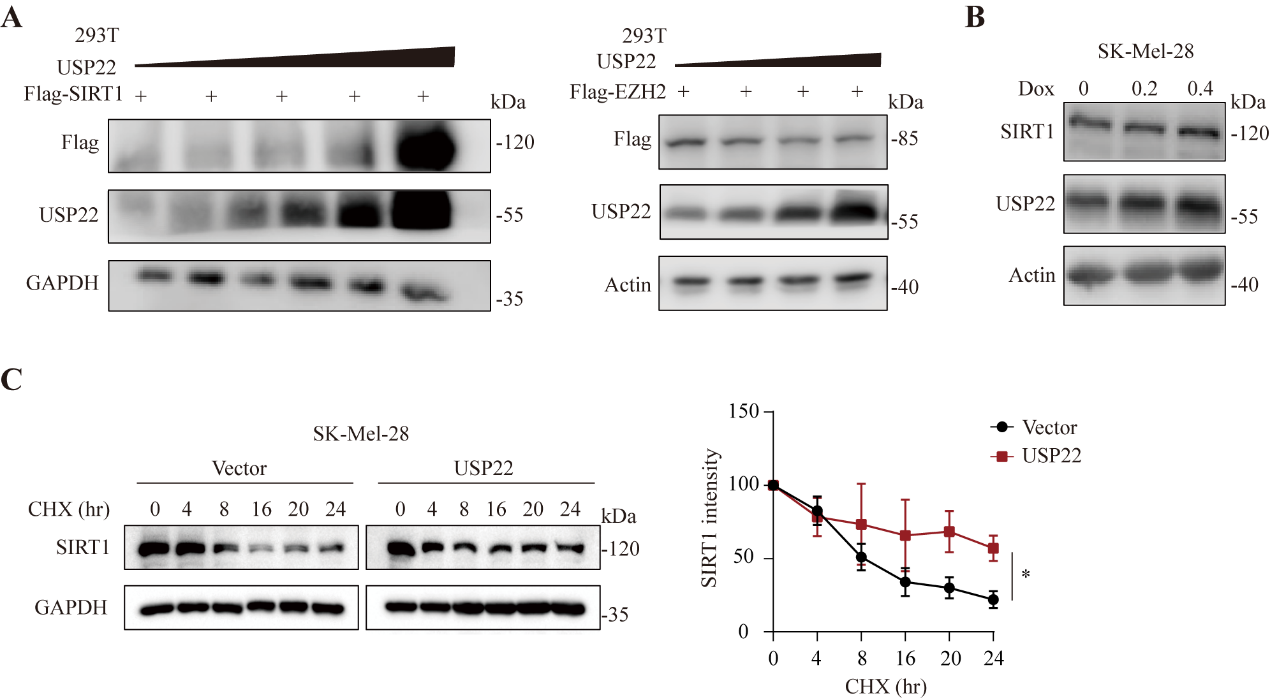
**

**Fig S4. USP22 stabilizes SIRT1 other than EZH2.**

**(A)** Immunoblot analysis of extracts of HEK293T cells transfected with increasing USP22 (wedge), Flag‐SIRT1 (0.5μg), Flag-EZH2 (0.5μg). **(B)** Western blotting showing SIRT1 and EZH2 protein expression in USP22‐inducible cells with doxycycline as indicated in SK-Mel-28 cells. **(C)** Protein lysates of control (Vector) and USP22-overexpressed (USP22) SK-Mel-28 cells were treated with cycloheximide (CHX) (100μg/mL) for the indicated time points, followed by immunoblotting with SIRT1. Two‐way ANOVA analysis was performed in (**C**). *p<0.05.

**
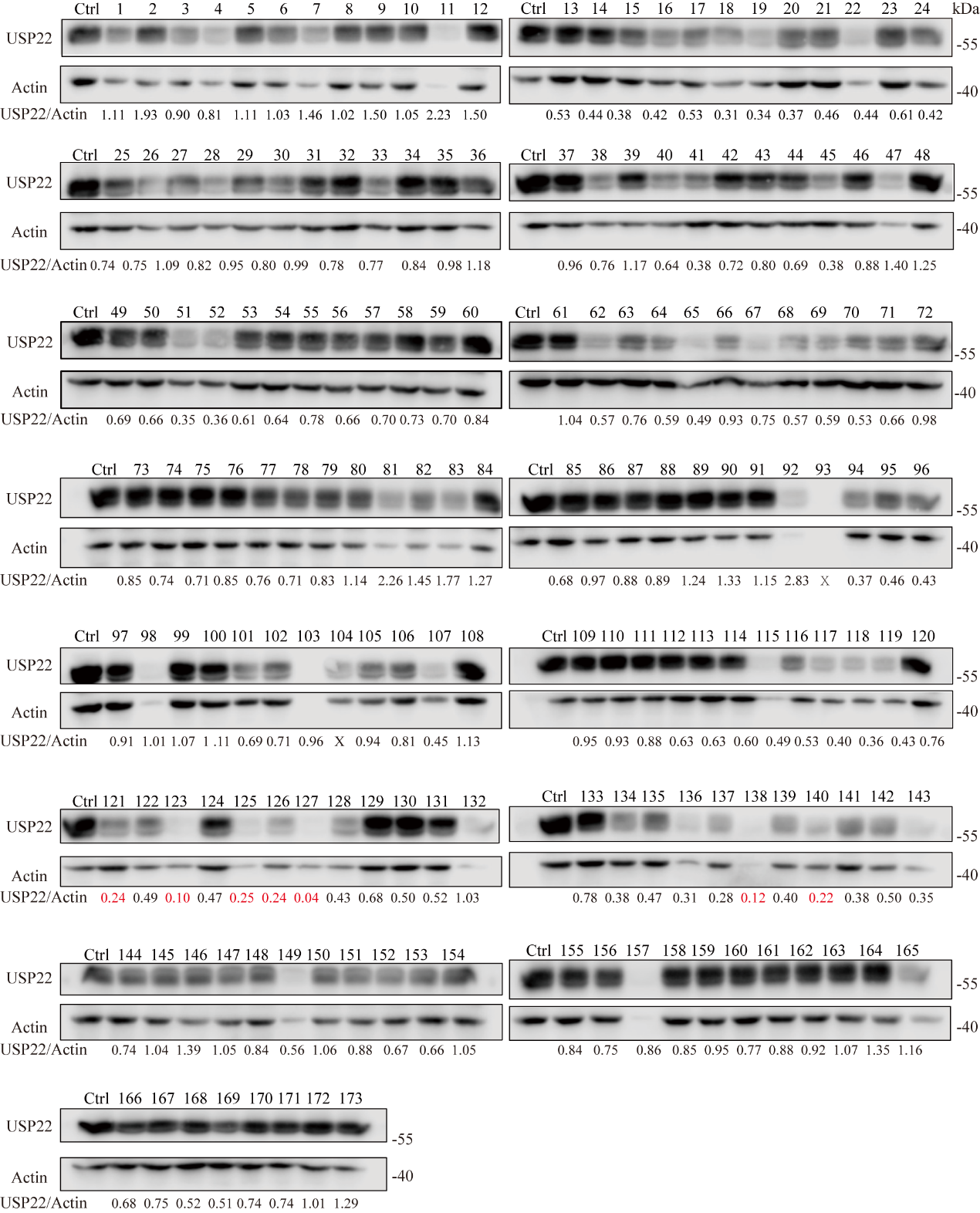
**

**Fig S5. FDA-drug screening identified USP22-targeting molecule.**

Western blotting‐based drug screening with concentration of 5μM for 24h identified 7 out of 173 compounds that decreased USP22 expression by 75%.

**
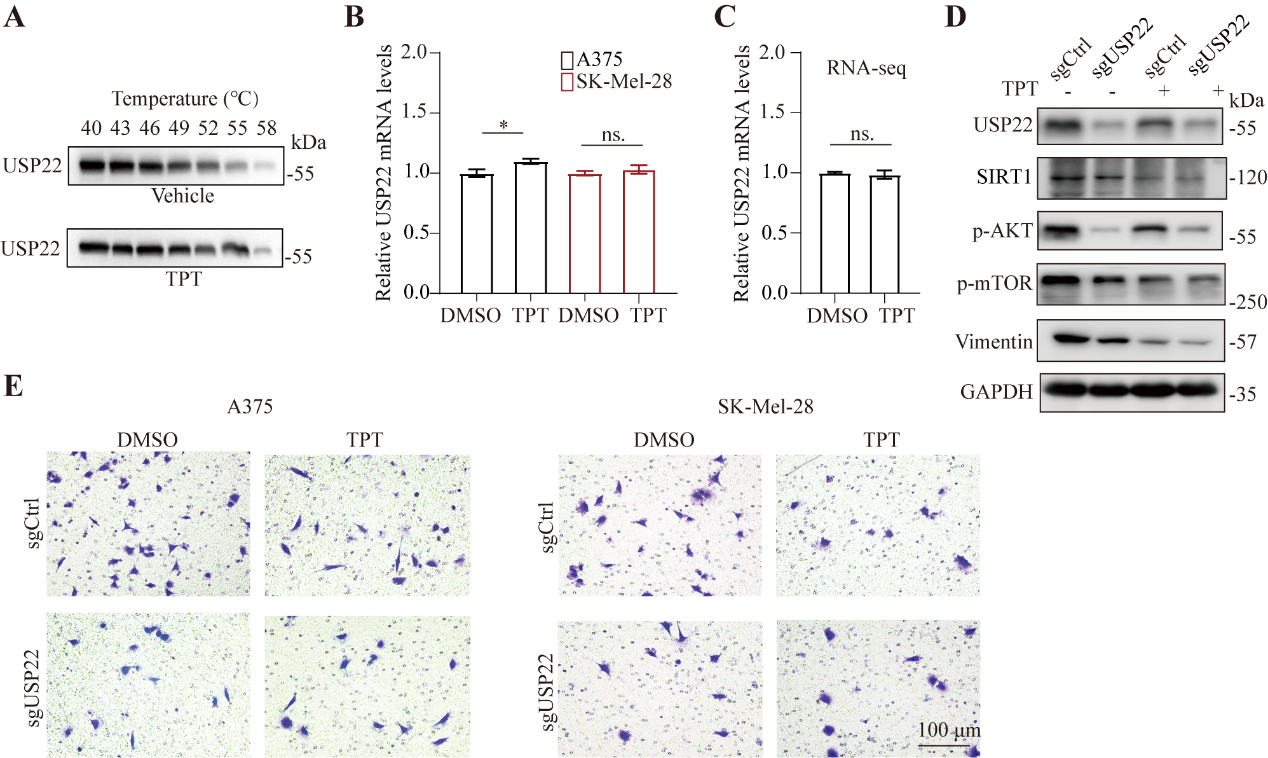
**

**Fig S6. Identification of USP22 inhibitor to suppress melanoma metastasis.**

(**A**) The cellular thermal shift assay (CETSA) of USP22 proteins for SK-Mel-28 cells treated with DMSO (Vehicle) or topotecan (TPT, 0.5 μM). **(B)** The mRNA levels of USP22 after treatment with DMSO (Vehicle) or topotecan (TPT, 0.5 μM) in A375 and SK-Mel-28 cells. **(C)** The mRNA levels of USP22 after treatment with DMSO or topotecan (TPT) based on RNA sequencing. **(D)** The protein levels of SIRT1, p-Akt, p-mTOR, and Vimentin in USP22 knockout (sgUSP22) or control (sgCtrl) A375 cells treated with 0.5uM topotecan for 24 h. **(E)** Transwell assay showing effect of topotecan (TPT, 0.5μM) on the migration of USP22 knockout (sgUSP22) A375 and SK-Mel-28 cells. Two‐tailed unpaired Student's t‐test was performed in (**B-C**). Ns, no significance. *p<0.05.

**
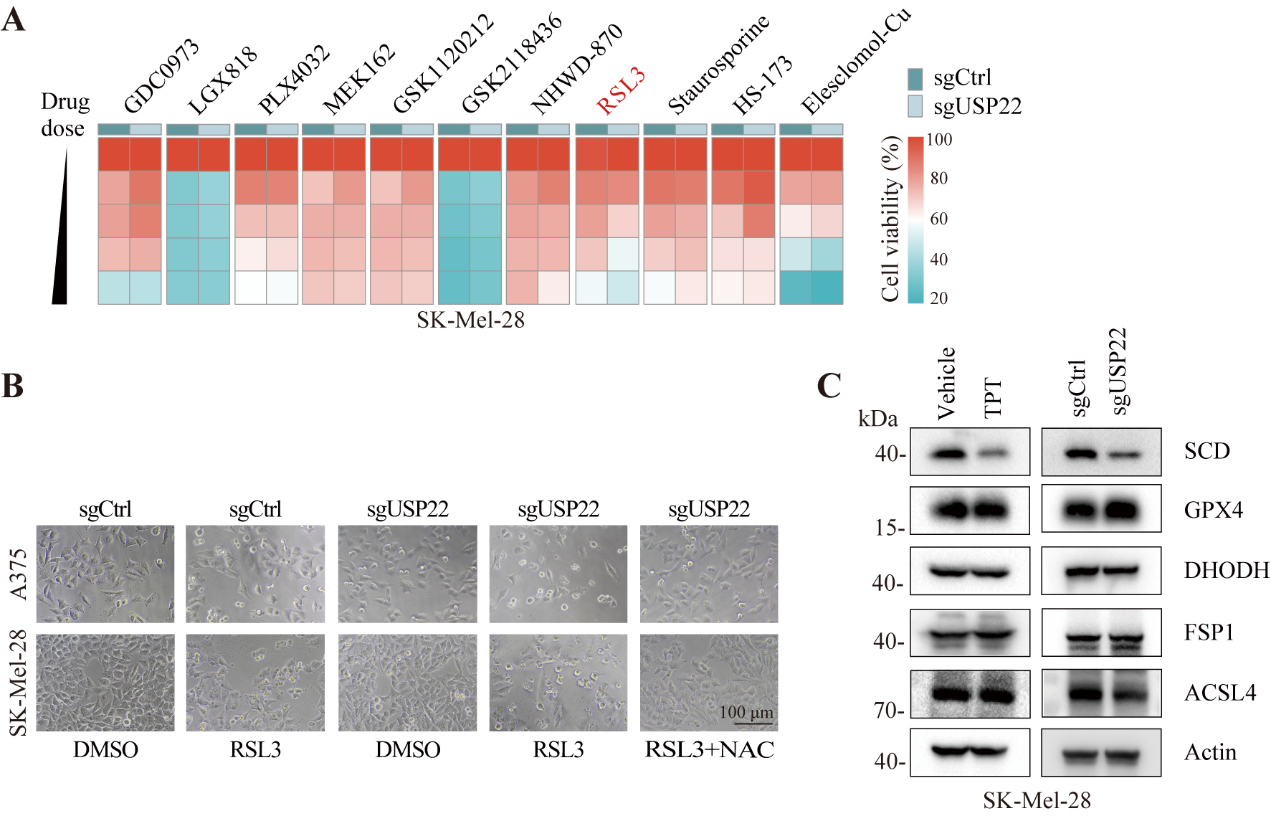
**

**Fig S7. USP22 silencing sensitized melanoma cells to RSL3-induced ferroptosis.**

**(A)** Heatmap showing cell viability of USP22-knockout (sgUSP22) and control (sgCtrl) SK-Mel-28 cells treated with BRAF inhibitors (LGX818, GSK2118436, PLX4032), MEK inhibitors (GDC0973, GSK1120212, MEK162) and BET inhibitor (NHWD-870), ferroptosis inducer (RSL3), apoptosis inducer (staurosporine), necroptosis inducer (HS-173), and cuproptosis inducer (Elesclomol-Cu). **(B)** Cell morphology of control (sgCtrl) and USP22-knockout (sgUSP22) A375 or SK-Mel-28 cells treated with RSL3 (2.5μM) alone or in combination with N-acetylcystiene (NAC, 200μM) as indicated. **(C)** Western blotting indicated the levels of ferroptosis-associated proteins (SCD, GPX4, DHODH, FSP1, and ACSL4) in SK-Mel-28 cells after treatment with topotecan (TPT, 0.5μM) or USP22 knockout (sgUSP22).

Table S1 primers for RT-PCR

| Gene symbol | Forward primer | Reserve primer |
| --- | --- | --- |
| USP22 | GGTCCCCTCACATCCCGTAT | TTGGGGTTGTTGGCCTTCTT |
| SNAIL  ZEB1  SLUG  ZEB2 | CTCTAATCCAGAGTTTACCTTC  AAAGATGATGAATGCGAGTC  CAGTGATTATTTCCCCGTATC  TTCCTGGGCTACGACCATAC | GACAGAGTCCCAGATGAG  TCCATTTTCATCATGACCAC  CCCCAAAGATGAGGAGTATC  GCCTTGAGTGCTCGATAAGG |
| CHAC1 | GTGGTGACGCTCCTTGAAGATC | GAAGGTGACCTCCTTGGTATCG |
| TFRC | ATCGGTTGGTGCCACTGAATGG | ACAACAGTGGGCTGGCAGAAAC |
| PTGS2 | CGGTGAAACTCTGGCTAGACAG | GCAAACCGTAGATGCTCAGGGA |

Table S2 The sequences of the siRNAs, shRNA and sgRNAs

|  | Forward（5'-3'） | Reserve（5'-3'） |
| --- | --- | --- |
| siUSP22 | GGAGAAAGAUCACCUCGAATT | UUCGAGGUGAUCUUUCUCCTT |
| siSIRT1 | GACUCAAGUUCACCAGAAAGATT | UCUUUCUGGUGAACUUGAGUCTT |
| siPTEN | GGAGGAUUAUUCGUCUUCUTT | AGAAGACGAAUAAUCCUCCT |
| shUSP22 | GACAACAAGTATTCCCTGTTT | |
| human-  sgUSP22 #1 | CTGCGTGGGCTGATCAACCT | |
| human-  sgUSP22 #2 | CTTTGTCATAGATGTAGTCC | |
| mouse-  sgUSP22 #1 | AGTAAATACCTCCGTACATC | |
| mouse-  sgUSP22 #2 | CAAGTCAAAGCGACACAACC | |

Table S3 Detailed information of tissue array panel

| No. | Age | Organ/  Anatomic Site | TNM | Stage | Tissue ID. | Type |  | (Positive Area %) | (Mean Density) | (Area Density) | H-Score |
| --- | --- | --- | --- | --- | --- | --- | --- | --- | --- | --- | --- |
| 1 | 44 | Skin | T4aN0M0 | IIB | Fvl070221 | Malignant | A1 | 14.8327 | 0.1397 | 0.020722 | 79.8109 |
| 2 | 41 | Skin | T4N0M0 | IIB | Fvl041282 | Malignant | A2 | 27.4636 | 0.0806 | 0.022124 | 69.7558 |
| 3 | 38 | Skin | T4bN0M0 | IIC | Fvl050429 | Malignant | A3 | 22.647 | 0.0946 | 0.02142 | 60.8897 |
| 4 | 57 | Skin | T4N0M0 | IIB | Fvl030086 | Malignant | A4 | 35.3904 | 0.1126 | 0.039852 | 113.1717 |
| 5 | 66 | Skin | T4bN0M0 | IIC | Dre080282 | Malignant | A5 | 47.6057 | 0.0789 | 0.037584 | 99.7799 |
| 6 | 84 | Skin | T4bN0M0 | IIC | Dre031701 | Malignant | A6 | 27.1343 | 0.0803 | 0.021797 | 73.0932 |
| 7 | 66 | Skin | T4bN0M0 | IIC | Dre031567 | Malignant | A7 | 28.5138 | 0.1139 | 0.032464 | 116.0677 |
| 8 | 52 | Skin | T4bN0M0 | IIC | Kin030086 | Malignant | A8 | 8.5754 | 0.1253 | 0.010744 | 42.1486 |
| 9 | 45 | Skin | T4bN0M0 | IIC | Kin020054 | Malignant | B1 | 25.3752 | 0.0969 | 0.024578 | 82.6313 |
| 10 | 40 | Skin | T4bN0M0 | IIC | Atc060179 | Malignant | B2 | 25.5947 | 0.0685 | 0.017523 | 109.4023 |
| 11 | 46 | Skin | T4bN0M0 | IIC | Sft060230 | Malignant | B3 | 15.857 | 0.1057 | 0.016761 | 85.3047 |
| 12 | 51 | Skin | T3bN0M0 | IIB | Kin060148 | Malignant | B4 | 10.8911 | 0.1525 | 0.016605 | 47.5708 |
| 13 | 36 | Skin | T4aN0M0 | IIB | Kin060059 | Malignant | B5 | 25.2361 | 0.0875 | 0.022072 | 154.1855 |
| 14 | 45 | Skin | T4bN0M0 | IIC | Kin030056 | Malignant | B6 | 39.8323 | 0.1017 | 0.040508 | 94.1629 |
| 15 | 66 | Skin | T4aN0M0 | IIB | Kin030031 | Malignant | B7 | 10.4812 | 0.1151 | 0.012063 | 51.8037 |
| 16 | 71 | Skin | T4bN0M0 | IIC | Kin090021 | Malignant | B8 | 31.4163 | 0.1198 | 0.037651 | 94.9448 |
| 17 | 38 | Skin | T4bN1M0 | IIIB | Kin020090 | Malignant | C1 | 10.8301 | 0.1051 | 0.011382 | 66.4494 |
| 18 | 42 | Skin | T4bN0M0 | IIC | Kin050012 | Malignant | C2 | 35.923 | 0.1332 | 0.047851 | 112.8382 |
| 19 | 38 | Skin | T4aN1M0 | IIIB | Sfb170134 | Malignant | C3 | 10.093 | 0.117 | 0.011806 | 58.2578 |
| 20 | 61 | Skin | T4aN0M0 | IIB | Kin090030 | Malignant | C4 | 24.7206 | 0.1239 | 0.030629 | 92.0562 |
| 21 | 49 | Skin | T4bN1M0 | IIIB | Kin080057 | Malignant | C5 | 23.4407 | 0.1397 | 0.032751 | 122.4465 |
| 22 | 41 | Skin | T4N0M0 | IIB | Kin080030 | Malignant | C6 | 33.6191 | 0.1543 | 0.051868 | 124.641 |
| 23 | 47 | Skin | T4aN0M0 | IIB | Kin030099 | Malignant | C7 | 26.8368 | 0.0995 | 0.026699 | 97.0458 |
| 24 | 49 | Skin | T4aN1M0 | IIIB | Sst050140 | Malignant | C8 | 36.5624 | 0.1233 | 0.045081 | 137.457 |
| 25 | 40 | Skin | T4bN0M0 | IIC | Kin060195 | Malignant | D1 | 31.085 | 0.1095 | 0.034026 | 87.3276 |
| 26 | 51 | Skin | T4aN0M0 | IIB | Kin050114 | Malignant | D2 | 26.9367 | 0.124 | 0.033407 | 106.0597 |
| 27 | 71 | Skin | T4bN0M0 | IIC | Sst030215 | Malignant | D3 | 45.1852 | 0.0969 | 0.043768 | 90.8702 |
| 28 | 60 | Skin | T4N0M0 | IIB | Kin060015 | Malignant | D4 | 32.6529 | 0.1098 | 0.035839 | 106.5411 |
| 29 | 61 | Skin | T4bN3cM0 | IIIC | Kin060180 | Malignant | D5 | 23.0103 | 0.1097 | 0.025246 | 82.1812 |
| 30 | 41 | Skin | T4aN0M0 | IIB | Kin060143 | Malignant | D6 | 30.5091 | 0.0823 | 0.025118 | 66.3057 |
| 31 | 45 | Skin | T4aN0M0 | IIB | Sst050082 | Malignant | D7 | 32.3228 | 0.1274 | 0.041179 | 130.7889 |
| 32 | 66 | Skin | T4bN0M0 | IIC | Kin020178 | Malignant | D8 | 27.9494 | 0.109 | 0.030452 | 79.5123 |
| 33 | 46 | Skin | T4bN1M0 | IIIB | Kin020154 | Malignant | E1 | 39.5581 | 0.075 | 0.029652 | 107.0087 |
| 34 | 73 | Skin | T3bN0M0 | IIB | Kin070084 | Malignant | E2 | 45.8899 | 0.1503 | 0.068955 | 114.8723 |
| 35 | 83 | Skin | T4bN0M0 | IIC | Kin060202 | Malignant | E3 | 36.4043 | 0.1196 | 0.043553 | 101.5855 |
| 36 | 52 | Skin | T4bN0M0 | IIC | Kin060158 | Malignant | E4 | 22.4104 | 0.0485 | 0.010873 | 111.1269 |
| 37 | 49 | Skin | T4N2M0 | IIIB | Kin060186 | Malignant | E5 | 46.6875 | 0.1774 | 0.082827 | 185.1109 |
| 38 | 54 | Skin | T4bN0M0 | IIC | Kin040053 | Malignant | E6 | 40.045 | 0.0796 | 0.031888 | 92.9499 |
| 39 | 80 | Skin | T4bN0M0 | IIC | Kin060124 | Malignant | E7 | 19.3796 | 0.1081 | 0.020956 | 77.4936 |
| 40 | 55 | Skin | T4bN0M0 | IIC | Kin060003 | Malignant | E8 | 16.8788 | 0.1322 | 0.022313 | 87.5406 |
| 41 | 25 | Skin | - | - | Kin15N009 | Normal | F1 | 8.9588 | 0.1648 | 0.014761 | 30.8427 |
| 42 | 21 | Skin | - | - | Kin15N008 | Normal | F2 | 11.2683 | 0.1197 | 0.013488 | 59.0039 |
| 43 | 21 | Skin | - | - | Kin15N007 | Normal | F3 | 12.0612 | 0.0972 | 0.011726 | 72.0817 |
| 44 | 35 | Skin | - | - | Kin08N011 | Normal | F4 | 14.5946 | 0.0954 | 0.013929 | 89.9819 |
| 45 | 30 | Skin | - | - | Kin08N004 | Normal | F5 | 13.6064 | 0.1046 | 0.014234 | 65.0707 |
| 46 | 40 | Skin | - | - | Kin08N002 | Normal | F6 | 17.2893 | 0.102 | 0.017634 | 96.8821 |
| 47 | 40 | Skin | - | - | Kin07N002 | Normal | F7 | 17.3834 | 0.1143 | 0.019876 | 88.9627 |
| 48 | 35 | Skin | - | - | Kin07N015 | Normal | F8 | 9.7354 | 0.1155 | 0.011247 | 68.3035 |
| 49 | marker |  |  |  |  |  |  | 27.3895 | 0.0883 | 0.024183 | 82.6447 |
